# Complementary treatment comparison for chronic pain management: A randomized longitudinal study

**DOI:** 10.1371/journal.pone.0256001

**Published:** 2021-08-06

**Authors:** Aminata Bicego, Justine Monseur, Alain Collinet, Anne-Françoise Donneau, Robert Fontaine, Dominique Libbrecht, Nicole Malaise, Anne-Sophie Nyssen, Mélissa Raaf, Floriane Rousseaux, Irène Salamun, Cécile Staquet, Sandrine Teuwis, Marco Tomasella, Marie-Elisabeth Faymonville, Audrey Vanhaudenhuyse

**Affiliations:** 1 Sensation and Perception Research Group, GIGA Consciousness, University of Liège, Liège, Belgium; 2 Cognitive Ergonomy and Work Intervention Department, University of Liège, Liège, Belgium; 3 Public Health Department, Biostatistics, University of Liège, Liège, Belgium; 4 Musicothérapie & Counselling, Boncelles, Belgium; 5 Interdisciplinary Algology Department, Hospital University of Liège, Liège, Belgium; Prince Sattam Bin Abdulaziz University, College of Applied Medical Sciences, SAUDI ARABIA

## Abstract

**Background:**

In chronic pain, it seems that the effect of cognitive-behavioral therapy (CBT) is boosted when it is combined with hypnosis. The aim of this study was to assess the efficacy of self-hypnosis combined with self-care (i.e., a type of CBT) compared to music/self-care, self-care and psychoeducation/CBT and to evaluate their long-term effects.

**Methods:**

An open label randomized clinical trial enrolled patients with chronic pain and was carried out at the University Hospital of Liège (Belgium). Patients were randomized into four groups: self-hypnosis/self-care, music/self-care, self-care, psychoeducation/CBT (7 monthly sessions of 2 hours). Two follow-up sessions were delivered at 6- and 12-month. Levels of pain, fatigue intensity, anxiety, depression, insomnia severity, disability, health locus of control, mental and physical quality of life and attitudes (control, disability, harm, emotion, medical cure, medication, solicitude) towards pain were assessed before and after the treatments, and at follow-up.

**Results:**

203 patients were randomized: 52 in self-hypnosis/self-care, 59 in music/self-care, 47 in self-care, and 45 in psychoeducation/CBT. No group effect was found. A significant time effect was showed. Directly after the treatment, all groups decreased in pain attitudes and physical quality of life. Perceived control increased. At 6-month, all patients kept their levels of physical quality of life and perceived control, and showed decrease in pain intensity, harm, emotion and medical cure. At 12-month, scores that had change previously remained ameliorated, a decrease in insomnia severity and an increase in internal locus of control were observed.

**Conclusions:**

The present findings are encouraging as they display long-term beneficial effects of complementary biopsychosocial-based treatments in chronic pain. It seems that patients continued to apply the learnt strategies as improvements were observed one year after the treatments had ended.

## Introduction

Pain is defined as “an unpleasant sensory and emotional experience associate with, or resembling that associated with, actual or potential tissue damage” in which sensory perceptions, emotional and cognitive processes intricately interact with one another [1]. Pain can be further classified into two categories: acute and chronic pain. The former acts as a protective and adaptive signal regarding one’s body integrity [2], while the latter loses its alarm signal and becomes an entity in its own right [3]. Chronic pain has been defined as a prolonged and persistent pain, lasting for more than 3 months beyond the expected healing period of tissue pathology [4]. Various psychological factors such as anxiety and depression have been linked to chronic pain [5–7]. A high prevalence of insomnia (53%) is also reported in chronic pain, with a positive correlation to anxiety and depression levels experienced by patients [8]. In addition, the chronic state of pain is often accompanied by changes in cognition, i.e., attitudes and beliefs towards pain, that are more consistent with patients’ persistent pain experience [5]. While some beliefs are considered to be adaptive (e.g., the fact that the emotional state influences pain), when such beliefs are altered, they could have a deleterious impact in the coping strategies that chronic pain patients use in their everyday life [9]. Furthermore, a study showed that chronic pain patients with an external locus of control, exhibited maladaptive coping strategies (e.g., helplessness) and were more prone to catastrophize and avoid increasing their activity in order to cope with pain [10]. Altogether, these studies demonstrate the complex and entangled processes at play in chronic pain and the need to rely on the biopsychosocial model in order to further understand and elaborate efficient treatments.

Complementary treatments are increasingly used by patients and are becoming more accepted in Europe. A study showed that 24.6% of people in Belgium used complementary treatments (e.g., acupuncture, homeopathy, hypnotherapy, massage therapy) during the 12 months prior to the study. Furthermore, compared to other chronic conditions (e.g., depression, breathing problems, diabetes) chronic pain conditions relied more on complementary treatments such as mind-body therapies (e.g., hypnosis) [11]. Nevertheless, even though interest in complementary therapies has increased in the past two decades, the evidence of their efficacy and effectiveness is still understudied [12]. Moreover, comparative effectiveness research often focuses on a specific effect of complementary treatment through standardized and ideal situations that are not representative of clinical practice [12]. In addition, increases in aging population will also probably augment the incidence of chronic pain. Therefore, the identification of effective treatments for pain management is important not only at the individual level but also at the societal one [13].

The goal of the Interdisciplinary Algology Department of the University Hospital of Liège (Belgium) is to assess non-pharmacological/complementary approaches in the management of chronic pain. Four complementary approaches—psychoeducation/cognitive-behavioral therapy (CBT), self-care, self-hypnosis combined with self-care (self-hypnosis/self-care) and specially composed music combined with self-care (music/self-care)—are proposed. The aim of psychoeducation is to enhance adaptive coping by informing the patient about pain’s physiological mechanisms, as well as the various consequences that can result from chronic pain such as emotional distress and alterations in cognition and behavior [14,15]. Psychoeducation is a CBT-based approach. The principal tool used is reformulation, which allows the patients to understand every step of the process and how it differs from what they had previously learned. Self-care is an approach that has been developed by one of the co-authors (M-E.F.) based on her clinical expertise with patient suffering from chronic pain. Self-care is an empowerment CBT-based intervention based on specific tasks, aiming to retrain the patient to be an actor in rather than an observer of his/her life condition. The distinctive feature of self-care is that this intervention is centered on emotions, cognition and behaviors, rather than on the pain problematic. During these group sessions, the therapist reviews the difficulties patients have had implementing the tasks given in the previous session. Self-hypnosis combined with self-care was also developed by M-E.F. This treatment consists of a combination of both the self-care technique and self-hypnosis learning. In addition to the tasks delivered to patients, self-hypnosis training is also introduced. The goal is to enable the patients to utilize hypnosis when they see fit. A full description of the hypnosis exercises is available in the Method section. Specially composed music was used as a control for the self-hypnosis exercises, with each piece of music composed by one of the co-authors (A.C.) based on the hypnotic voice and script of the hypnosis exercises.

These complementary approaches have shown encouraging results in the management of chronic pain [16,17]. More recently, studies have suggested that when hypnosis is combined with CBT approaches, it boosts the CBT-based treatment’s effectiveness [18]. Therefore, the aim of this randomized longitudinal study was to assess whether self-hypnosis/self-care (self-care being a type of CBT) is more effective than music/self-care, self-care alone and psychoeducation/CBT. First, we hypothesize that the patients in the self-hypnosis/self-care group will have greater improvement in all outcomes compared to the patients who received music/self-care, self-care alone and psychoeducation/CBT. Second, we hypothesize that the beneficial effects obtained directly after the end of the treatment would last at least to the 6- and 12-month follow-ups. This study is of clinical relevance because if hypnosis effectively enhances CBT-based approaches it means that clinicians will be able to propose cost-effective complementary treatments to patients. Furthermore, it will provide new insight to clinical researchers and allow future studies to be carried out in order to better understand the mechanisms underlying the enhancement of CBT-based approaches via hypnosis.

## Materials and methods

### Trial design

The current study was a four-arm parallel-design randomized controlled trial. Due to the type of intervention, neither patients nor therapist could be blinded, rendering this trial an open-labelled one. No changes in the Methods were implemented after trial commencement.

The procedure included (1) a screening phase in which a medical doctor specialized in pain management (i.e., an algologist) elaborated a pain diagnosis; (2) a baseline pre-treatment examination of patients’ health characteristics conducted by a nurse with questionnaires (T1); (3) a multidisciplinary meeting where the clinical team proposed a multidisciplinary approach and randomized patients into the treatment groups (carried out with the randomization function of Microsoft Excel); (4) a patient meeting with a pain specialist who proposed the treatment allocation; (5) a treatment phase (7 months); (6) a post-treatment assessment of patients’ health characteristics using the same questionnaires as in the pre-treatment phase (T2); two follow-up sessions and completion of the same questionnaires at (7) 6 months and (8) 12 months after the end of treatments. Each approach consisted of 7-monthly sessions of 2 hours plus two follow-up sessions of 2 hours (6 and 12 months). Each group session was composed of 8 patients. The functioning of these treatments was previously published in [19,20].

### Ethical considerations

The study was approved by the Ethics Committee of the Medical School of the University of Liège, Belgium (reference number: 2015/85) and retrospectively registered on ClinicalTrials.gov (NCT04261530; Treatment Comparison in Chronic Pain: a Randomized Study) on October 21^st^ 2019. The authors confirm that all ongoing and related trials for this intervention are registered. The full trial protocol is available in S1 and S2 Files. All participants gave written informed consent to participate in the study.

### Randomization

The randomization was carried out with the randomization function of Microsoft Excel by co-author A.V. This function was used for each participant. The multidisciplinary team all contributed to the recruitment and enrollment of the patients.

### Participants

From March 17^th^ 2015 to December 5^th^ 2017, chronic pain patients who attended the Interdisciplinary Algology Department of the University Hospital of Liège (Belgium) were recruited. The study started on March 17^th^ 2015 and ended on February 6^th^ 2020. Out of the 607 patients who attended the department, seven (1.1%) patients were not interested in the study, 23 (3.8%) did not complete the T1 questionnaires, one (0.2%) had another health issue (preventing her from attending treatments) and one other (0.2%) could not attend the group sessions because of for organizational purposes. Three hundred seventy-two (61.3%) patients could not be randomized as they came to the department with the specific demand to learn self-hypnosis/self-care, and thus their data were not considered in the present study. Ultimately, a total of 203 (33.4%) patients were included in this study and randomized into four groups: psychoeducation/CBT (n = 45), self-care (n = 47), self-hypnosis/self-care (n = 52), and music/self-care (n = 59). Patients were eligible for inclusion if they were: at least 18 years old, fluent in French, and had a diagnosis of chronic pain (pain > 3 months). Exclusion criteria were: psychiatric disorders (schizophrenia, psychosis, borderline with prolonged dissociation episode), drug addiction, and alcoholism.

### Interventions

*Psychoeducation/CBT* was conducted by two psychologists specialized in pain management (N.M. and I.S.). This is a cognitive-behavioral-based approach that aims to improve the self-management capacities and coping processes of patients with regard to their chronic pain. This intervention involved supportive group discussions. These discussions aimed to empower the patients to become active participants in their own treatment, and to provide them with a comprehensible model of pain mechanisms, an understanding of the rationale for pharmacological, physical and psychological therapy, and an acceptable rationale for making lifestyle changes. During these discussions, the psychologists gave a presentation on the understanding of the problems underlying chronic pain, on the biopsychosocial approach, and on the benefits of patient empowerment. Sessions also included open discussions on several themes, such as the specificities of chronic pain, the psychological factors linked to it, personal issues, attitudes and beliefs, and several tips on coping with the features of chronic pain. The aim of psychoeducation/CBT is thus to enhance adaptive coping by informing the patient about physiological pain mechanisms as well as the interactions between emotional distress, altered cognition and behaviors.

*Self-care* was conducted by three psychologists (N.M., I.S., and A.V.) and by an algologist (M-E.F.). The self-care approach is an intervention developed by one of the co-authors (M-E.F.) based on her clinical experience with chronic pain patients. Similarities with Acceptance and Commitment Therapy (ACT) can be found in self-care. Indeed, its goal is not to suppress pain but rather to get the patients to tailor responses to their symptoms according to their own goals [21]. Furthermore, the principle of self-care is to teach the patient to take care of themselves in their everyday life through concrete tasks. The objective is to place the patient in an active role in the management of their chronic pain, as well as to reactivate and amplify the patient’s awareness of the positive experiences encountered every day. All of the proposed tasks focus on the patients’ general well-being rather than on the problem of pain. The following exercises were proposed: adjusting expectations of self, changing the patient’s self-narrative, strengthening self-esteem, observing and readjusting the social roles of the patient, identifying limits and needs, identifying situations and feelings of powerlessness, accepting the impossibility of controlling everything, differentiating self from illness. These exercises were explained and discussed in groups and were given as homework. Patients were invited to keep a diary in which they could keep track of the facilities and difficulties of daily task application that would then be discussed during each new session.

*Self-hypnosis/self-care* was conducted by the four previously mentioned therapists (N.M., I.S., A.V., and M-E.F.), all of whom are specialized in clinical hypnosis. The aim of this treatment was the same as that mentioned above. In addition to the tasks, at the end of each session, a 20-minute hetero-hypnosis exercise was conducted by the therapist with the group of patients. Each patient also received individual CDs containing the hypnosis exercise from the session, and they were invited to perform the exercise on a daily basis in order to attain self-hypnosis. The first session was an introduction in which the therapist explained hypnosis and carefully corrected myths and misunderstandings patients had towards hypnosis. The seventh session was also an informative session where the therapist summarized the content of the six previous sessions and, talked about chronic pain its neurophysiology and other mechanisms inherent to this condition. For all hypnosis exercises, the therapist first invited the patient to take a moment to find a comfortable position, before continuing with the induction: visual fixation and/or breathing attention focalization. Five different hypnosis exercises were given. The first exercise, named “Soothing White Clouds”, included suggestions about relaxation, positive body sensations and an invitation to observe a sunrise and a beautiful landscape while relaxing in a white cloud chair [22]. The second exercise was named “Heaven of Peace”, in which suggestions of a safe place were delivered. The third exercise, “Land of Dreams”, was centered on healing sleep suggestions and invited patients to observe a pleasant trail that led to a dreamy land. The fourth exercise “Color-Pain” was based on analgesia suggestions, with patients invited to focus on the different pain intensities and locations they felt in their body and to select a color according to the pain intensity. Then, patients were invited to visualize the fading of colors, hence suggesting analgesia. The fifth exercise, “Self-protection”, was also based on analgesia suggestions. The patients were invited to visualize an analgesic glove that could spread the analgesic effect to whatever body part needed it. The aim of these exercises was to increase comfort, sleep quality, and to decrease pain sensations. During the follow-up sessions, two more exercises were delivered. The first one suggested a sensation of lightness in the arm, named “Travel Light”. The second, named “Stories and Metaphores”, was centered on problem solving via suggestions of metaphors. Patients were invited to find an object that would represent a problem and were then invited to alter the object in order to improve this problem.

*Music/self-care* was conducted by the four previously mentioned therapists (N.M., I.S., A.V., and M-E. F.). The aim and the procedure were the same as in the “self-hypnosis/self-care” group, except no hypnosis exercise was given. Instead, at the end of each session, patients were invited to listen to a specially composed musical melody for 20 minutes. Each piece of music was specially composed by a certified music therapist (A.C.) according to the hypnosis exercise. It followed the rhythm of the hypnotic voice and the music therapist chose sounds that were representative of the metaphors utilized in the hypnotic script. Five CDs were given to each patient and they were asked to listen to them on a daily basis. Two additional CDs were given during follow-up sessions. Each group consisted of 8 patients. Music as a control for hypnosis was previously used and shown to be effective [23,24].

### Outcomes

Data collection at the four time points (T1, T2, T3, T4), followed the Initiative in Methods, Measurements and Pain Assessment in Clinical Trials (IMMPACT) recommendations [25]:

#### General information

Medical and sociodemographic information of patients such as sex, age, educational level (i.e., higher degree obtained), occupational status, diagnosis (more than one diagnosis per patient possible), and pain duration. We also recorded if patients were part of the agreement between the Interdisciplinary Algology Department and National Institute for Health and Disability Insurance of Belgium (NIHDI) (named “convention” in this study). This agreement allowed a predetermined number of chronic pain patients to receive a multidisciplinary pain diagnosis and an adapted treatment program while the cost of clinical workup and treatment were directly reimbursed to the Interdisciplinary Algology Department by the NIHDI. The delay between the first contact of the patient with the Interdisciplinary Algology Department and the start of treatment was also collected via the medical record of the patient.

*Numerical Rating Scales (NRS)* were used in order to assess pain and fatigue intensity, as subjectively perceived by the patients, on a scale ranging from 0 (no pain/fatigue) to 10 (pain/fatigue as intense as you could imagine) [26,27].

*The Hospital Anxiety and Depression Scale (HADS)* was used to examine the levels of anxiety and depression subjectively perceived by patients. HADS is a 14-items self-report screening and contains two 7-items subscales for anxiety and depression, with a total score for each ranging from 0 to 21. The higher the score, the higher the symptoms [28]. The French version of HADS was used, having a good internal consistency of 0.81 for the anxiety subscale and 0.78 for the depression subscale [29].

*The Insomnia Severity Index (ISI)* was administered in order to measure insomnia-related difficulties. It comprises 7 items that examine the severity of sleep onset, sleep maintenance, satisfaction with current sleep, interference of sleep difficulties with daily functioning, and noticeability of impairment due to sleep alterations [30]. The French version of the ISI, which has a good internal consistency of 0.92 [31] was administered.

*The Pain Disability Index (PDI)* was used to assess the repercussions of the interference of pain in the patients’ ability to engage in various activities. This is a self-reported scale rating the extent of disability in a target activity on a range from 0 to 10. PDI contains seven categories of life activities: family/home, responsibility, recreation, social activity, occupation, sexual behavior, self-care and life support activity. The higher the score attained, the higher the perceived disability [32]. The French version of the PDI which has a good internal consistency of 0.83 [33], was administered.

*The Multidimensional Health Locus of Control Scale (MHLC)* was used to measure the type of locus patients had. The scale is divided into three sub-scales [34]. The Internal Health Locus of Control (IHLC) represents an internal locus of control, meaning that if one scores high on this subscale, one views health problems as a consequence of one’s behaviors. The Powerful Other Health Locus of Control (PHLC) corresponds to an external locus of control. A high score on this subscale means that one attributes his/her health problems to an external person. The Chance Health Locus of Control (CHCL) reflects an external locus of control assigned to chance. The French version of the MHLC was administered. The IHLC, PHLC and CHLC have internal consistency scores of 0.82, 0.84 and 0.61 respectively [35].

*The Survey of Pain Attitude-35 (SOPA-35)* was administered to understand the attitudes and beliefs chronic pain patients endorsed. This scale is composed of 7 subscales. Harm measures the belief that hurt means physical injury. Disability assesses the belief that one is disabled by pain. Medication examines the belief that the best treatment is medication. Solicitude assesses the belief that it is the other’s responsibility to assist one with his/her pain. Emotion measures the belief that emotions influence the pain experience. Medical cure assesses the belief that it is the doctor’s duty to relieve one’s pain. Control measures the amount of control one believes one has over the pain experience. There is a specific score to each subscale—the higher the score, the more the patient endorses the belief [36].

*The Short Form-36 (SF-36)* assesses global quality of life. This is a 36-item scale that comprises 8 subscales, from which 2 summary scores emerge: mental (MCS) and physical (PCS). The higher the score, the better the mental/physical quality [37]. The French version of the SF-36 was administered. The consistency was assessed for the 8 subscales and ranged from 0.76 to 0.92 [38].

*The Patient Global Impression of Change (PGIC)* was used to assess the patient’s belief about the efficacy of the treatment. It is a single item scale assessed through a 7-point scale. The higher the score the worse the improvement [39]. Content validity documentation of the PGIC is available at https://eprovide.mapi-trust.org/instruments/patient-global-impressions-scale-change-improvement-severity.

We used French validation for all questionnaires except the SOPA-35 and the pain and fatigue NRS. No changes to trial outcomes were carried out after the trial started.

### Sample size

The sample size calculation was based on a repeated measure, between factor ANOVA a difference in mean scores using power calculation. Alpha was set at 0.05, power at 90% and the standardized effect size at 0.3. According to this analysis, 26 patients were required in each group for a total of 104 patients. According to the literature [40] and our clinical expertise, dropout rates commonly reach 40% in chronic pain management studies. We thus recruited approximatively 50 patients per group of treatment.

### Statistical analyses

First, descriptive statistics were conducted. Qualitative variables were expressed with count and percentage. The normality of quantitative variables was examined numerically by comparing mean and median, graphically investigated histogram and quantile-quantile plots. A Shapiro-Wilk normality test completed this investigation of normality. If normality was assumed for the distribution of the quantitative variable, means and standard deviations were reported. Reversely, medians and interquartile ranges were presented. In order to detect potential confounding factors, baseline characteristics were compared between the 4 groups using *χ*^2^ test for qualitative variables and one-way ANOVA or its non-parametric equivalent Kruskall-Wallis test for quantitative variables. Then, repeated-measure ANOVA group-by-time was applied to examine the presence of significant differences in the mean scores of the 18 dependent variables (pain and fatigue NRS scores, HADS, ISI, PDI, MHLC, SOPA-35, SF-36, PGIC) across 4 time points and 4 treatment groups, (psychoeducation/CBT, self-care, self-hypnosis/self-care, music/self-care). Self-hypnosis/self-care was the reference category. Normality and homogeneity of the variance of the residuals were assessed graphically to check assumptions of models. When necessary, the models were adjusted for detected potential confounding factors. In case of group differences, multiple pairwise comparisons were performed. In addition, multiple comparisons using Bonferroni as correction (p = 0.01) method were realized between T1 and T2, T1 and T3, and T1 and T4. Results were considered significant at the 5% critical level (two-tailed p < 0.05). An Intention-to-Treat (ITT) procedure was used in order to included subjects who dropped out of the study [41]. To that mean, we applied last observation carried forward. The analyses were conducted with the software R [42] version 3.6.1.

## Results

**Fig 1:**
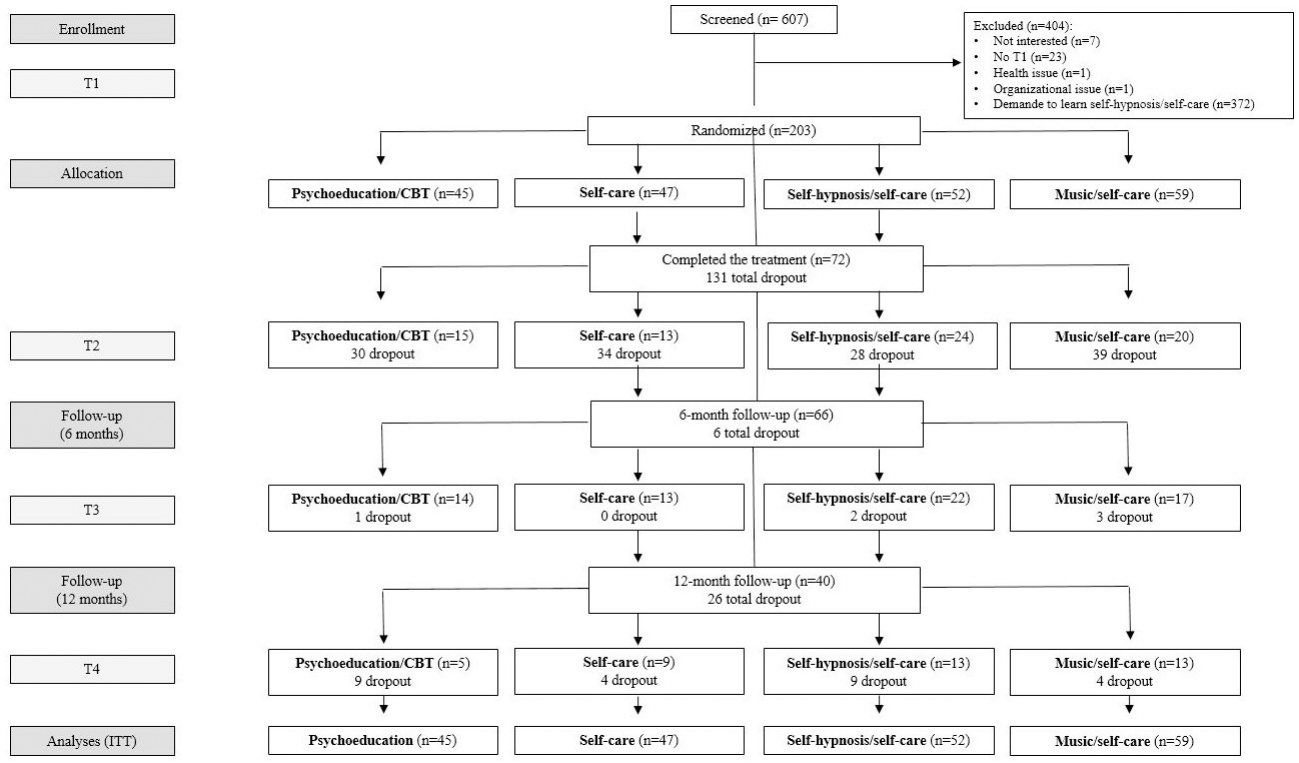
Flow chart. Number of patients included in the study and number of patients who dropped out. ITT: intention-to-treat.

As depicted in Fig 1, out of the 203 patients, 72 (35.5%) completed the treatment (T2) and 131 (64.5%) dropped out. Sixty-six (32.5%) patients completed the 6-month follow-up (T3), meaning that 6 patients dropped out between T2 and T3. Forty (19.7%) patients completed the 12-month follow-up (T4), meaning that 26 patients dropped out between T3 and T4. Considering the treatment groups separately, 45 (22.2%) were randomized in the psychoeducation/CBT group, 47 (23.1%) in the self-care group, 52 (25.6%) in the self-hypnosis/self-care group and 59 (29.1%) in the music/self-care group. Fifteen (33.3%) patients completed the treatment (T2) in the psychoeducation/CBT group, 13 (27.6%) in the self-care group, 24 (46.1%) in the self-hypnosis/self-care group, and 20 (33.9%) in the music/self-care group. At the 6-month follow-up (T3), the number of patients completing the program was 14 (31.1%) in the psychoeducation/CBT group, 13 (27.6%) in the self-care group, 22 (42.3%) in the self-hypnosis/self-care group, and 17 (28.8%) in the music/self-care group. This decrease continued at the 12-month follow-up (T4), with 5 (11.1%) patients in the psychoeducation/CBT group, 9 (19.1%) in the self-care group, 13 (25%) in the self-hypnosis/self-care group, and 13 (22%) in the music/self-care group.

### Descriptive analyses

The mean age of patients was 46.7 years (±8.8) and, 182 (89.6%) patients were women. As presented in Table 1, no statistical differences were observed between groups for age, sex, educational level, occupational status, convention, pain duration, and diagnosis of chronic pain syndrome, back pain, headaches, osteoarthritis, mixed pain, neuropathic pain, visceral pain and other etiologies (i.e., tinnitus, dysfunctional somatic syndrome, stiff neck) and delay onset of treatments. Statistical differences across groups were observed for fibromyalgia [5 (11.1%) patients in the psychoeducation/CBT group, 20 (42.6%) in the self-care group, 14 (26.9%) in the self-hypnosis/self-care group, and 11 (18.6%) in the music/self-care group; p = 0.003] and polyalgia diagnoses [25 (55.6%) in the psychoeducation/CBT group, 12 (25.5%) in the self-care group, 13 (25%) in the self-hypnosis/self-care group, and 18 (30.5%) in the music/self-care group; p = 0.004].

**Table 1:** Patients baseline characteristics globally, and within each group.

	All (N = 203)		Psychoeducation/CBT (n = 45)		Self-care (n = 47)		Self-hypnosis/self-care (n = 52)		Music/self-care (n = 59)		p-value
	N		n		n		n		n		
**Factors**											
**Age**, years, mean±SD	203	46.7±8.77	45	48.22±8.65	47	44.87±8.16	52	48.55±8.42	59	45.37±9.29	0.77
**Sex**, n (%)	203		45		47		52		59		
Female		182 (89.6)		42 (93.3)		41 (87.2)		47 (90.4)		52 (88.1)	0.76
Male		21 (10.4)		3 (6.7)		6 (12.8)		5 (9.6)		7 (11.9)	
**Education level**, n (%)	198		44		46		51		57		
Low (≤ 6 years)		24 (12.1)		8 (18.2)		6 (13.1)		2 (3.9)		8 (14)	
Intermediate (12 years)		137 (69.2)		31 (70.4)		30 (65.2)		41 (80.4)		35 (61.4)	0.19
High (≥ 15 years)		37 (18.7)		5 (11.4)		10 (21.7)		8 (15.7)		14 (24.6)	
**Occupational status**, n (%)	203		45		47		52		59		
Employed		42 (20.7)		11 (24.4)		9 (19.1)		9 (17.4)		13 (22)	
Unemployed/disable		146 (71.9)		27 (60)		35 (74.5)		40 (76.9)		44 (74.6)	0.27
Retired		5 (2.5)		3 (6.7)		0 (0.0)		2 (3.8)		0 (0.0)	
Other		10 (4.9)		4 (8.9)		3 (6.4)		1 (1.9)		2 (3.4)	
**Convention**, n (%)	203	97 (47.8)	45	23 (51.1)	47	24 (51.1)	52	24 (46.2)	59	26 (44.1)	0.85
**Diagnosis**, n (%)	203		45		47		52		59		
Chronic pain syndrome		30 (14.8)		5 (11.1)		10 (21.3)		9 (17.3)		6 (10.2)	0.34
Fibromyalgia		50 (24.6)		5 (11.1)		20 (42.6)		14 (26.9)		11 (18.6)	0.003*
Polyalgia		68 (33.5)		25 (55.6)		12 (25.5)		13 (25)		18 (30.5)	0.004*
Back pain		30 (14.8)		7 (15.6)		6 (12.8)		9 (17.3)		8 (13.6)	0.91
Headaches		5 (2.5)		1 (2.2)		0 (0.0)		1 (1.9)		3 (5.1)	0.39
Osteoarthritis		1 (0.5)		0 (0.0)		0 (0.0)		1 (1.9)		0 (0.0)	0.40
Mixed pain		1 (0.5)		1 (2.2)		0 (0.0)		0 (0.0)		0 (0.0)	0.31
Neuropathic pain		8 (3.9)		2 (4.4)		3 (6.4)		2 (3.8)		1 (1.7)	0.66
Visceral pain		5 (2.5)		0 (0.0)		0 (0.0)		2 (3.8)		3 (5.1)	0.21
Other etiologies		38 (18.7)		7 (15.6)		8 (17)		9 (17.3)		14 (23.7)	0.69
**Pain duration** in years, median [interquartile]	203	5 [3–10]		5 [3–10]	47	4 [2.5–7]	52	5 [2–9.5]	59	5 [3–11]	0.39
**Delay onset of treatment** in months, median [interquartile]	203	4 [3–5]	45	4 [3–5]	47	3 [2–5]	52	5 [2–6]	59	3 [3–5]	0.37

SD: Standard deviation; CBT: Cognitive-behavioral therapy.

No significant difference was observed for all questionnaire variables across the four groups at pre-treatment (T1) (p>.05). Summary of the 19 variables at each time point and in each group are presented in Table 2.

**Table 2:** Mean and standard deviation (±SD) of the variables measured at T1 (baseline), T2 (post-treatment), T3 (6-month follow-up) and T4 (12-month follow-up) after application of last observation carried forward method.

	Psychoeducation/CBT(n = 45)					Self-care(n = 47)					Self-hypnosis/self-care (n = 52)					Music/self-care (n = 59)				
	n	T1	T2	T3	T4	n	T1	T2	T3	T4	n	T1	T2	T3	T4	n	T1	T2	T3	T4
**Questionnaires** Mean±SD																				
**Pain Intensity** (NRS)	45	6.04±1.46	5.88±1.65	6.11±1.52	6.04±1.65	47	5.74±1.51	5.42±1.66	5.51±1.82	5.57±1.37	52	6.19±1.45	5.88±1.62	5.75±1.91	5.92±1.86	59	6.22±1.30	5.91±1.45	5.83±1.44	6±1.37
**Fatigue Intensity** (NRS)	45	7.46±1.58	7.13±1.85	7.37±1.66	7.24±1.79	47	7.461.69	7.3±1.79	7.29±1.94	7.34±1.73	52	7.23±2.26	7.21±2.19	7.19±2.07	7.25±2.11	59	7.28±1.95	7±2.16	7.16±2.24	7.27±2.09
**Hospital Anxiety and Depression Scale**	45					47					52					59				
Anxiety		13.04±4.19	12.20±4.37	11.84±4.63	12.02±4.46		12.44±4.35	12.27±4.28	12.19±4.13	12.14±4.34		12.09±4.06	11.86±4.24	11.98±4.64	11.90±4.38		12.45±4.20	11.96±4.53	12.05±4.52	11.83±4.70
Depression		11.06±3.38	10.28±3.27	10.33±3.66	10.17±3.59		11.80±3.92	11.36±3.88	11.14±3.91	11.38±3.82		10.07±4.11	9.96±4.29	10.13±4.24	9.76±4.34		10.79±3.92	10.08±3.82	10.01±4.26	9.88±4
**Insomnia Severity Index**	45	20.26±8.49	17.84±6.32	18.35±6.08	18.22±6.33	46	20.13±8.14	19.06±8.26	18.82±8.34	19.11±8.55	52	21.76±9.81	20.78±9.95	20.40±9.71	19.94±9.94	58	20.06±7.52	19.15±7.95	19.41±8.10	19.60±7.95
**Pain Disability Index**	45	43.82±10.44	41.48±12.08	40.84±12.38	40.84±12.64	47	41.72±13.33	40.46±14.35	40.08±13.67	39.63±14.08	52	43.36±11.83	41.13±13.38	41.55±14.63	41.80±14.05	59	44.84±12.46	42.50±13.71	43.08±13.38	43±12.84
**Multidimensional Health Locus of Control**	44					46					51					59				
CHLC		13.13±2.73	13.18±2.53	13.25±2.68	13±2.90		13.30±2.35	13.39±2.33	13.43±2.21	13.43±2.16		12.77±2.78	12.56±2.74	12.22±2.71	12.56±2.77		13.18±2.64	13.06±2.36	13.03±2.31	13.05±2.12
IHLC		13.43±2.62	13.75±2.52	13.77±2.74	13.84±2.86		12.91±2.91	13.32±2.92	13.47±3.14	13.34±2.93		13.43±3.81	13.70±3.79	13.82±4.01	14.04±4.01		13.40±2.82	14.06±2.96	14.20±3.15	13.96±2.93
PHLC		11.97±2.36	12.11±2.16	11.63±2.36	11.63±2.45		11.52±2.45	11.63±2.67	11.50±2.63	11.54±2.76		11.47±2.63	11.52±2.53	11.47±2.46	11.50±2.52		11.74±3.14	11.76±2.98	12.03±2.85	12.03±3.02
**Short Form-36**	45					47					52					59				
MCS		23.50±10.85	24.98±10.83	25.20±11.80	25.48±11.14		23.56±12.51	24.95±12.21	25.44±12.55	25.39±12.89		24.79±11.75	24.44±12.05	24.89±13.58	25.40±13.55		24.09±12.42	26±13.14	26.62±13.66	26.47±13.18
PCS		29.74±6.45	31.21±7.04	30.48±6.32	30.73±6.42		31.43±6.98	32.89±6.84	32.49±7.48	32.17±7.29		29.40±7.47	31.64±8.32	31.92±8.22	32.19±8.74		29.62±6.52	30.78±7.27	29.60±6.37	29.65±6
**Survey of Pain Attitudes-35**	45					47					52					59				
Control		6.06±3.75	6.88±3.94	6.82±4.22	6.73±4.27		5.46±3.71	6.25±4.22	6.74±4.46	6.57±4.19		5.92±3.78	6.84±4.34	7.25±4.90	7.59±5.25		6.57±3.65	7.77±4.36	7.55±4.27	7.37±4.02
Disability		14.60±3.25	13.82±3.47	14.08±3.42	13.95±3.57		13.95±3.32	13.25±3.42	13.14±3.50	13.27±3.70		15±3.63	14.75±4.01	14.264.45	14±4.57		14.52±3.65	13.49±3.85	13.79±4.05	13.76±4.11
Harm		10.86±3.91	9.80±3.70	9.93±3.82	9.95±3.87		10.19±3.89	9.19±3.73	9.61±3.55	9.51±3.62		10.19±3.53	9.15±3.53	8.92±3.77	8.82±4.06		10.47±3.50	9.98±3.67	9.81±3.86	9.74±3.89
Emotion		11.73±4.28	12.31±3.69	12.51±3.57	12.46±3.55		11.89±4.88	12.82±5.21	12.53±5.13	12.89±4.83		11.59±4.81	12.71±4.84	12.84±4.91	12.88±4.93		11.93±5.24	12.72±4.87	12.71±5.03	12.72±4.95
Solicitude		11.04±3.74	10.55±3.88	10.08±4.31	10.02±4.38		8.63±4.53	8.70±4.74	8.68±4.58	8.91±4.59		8.50±5.52	8.38±5.09	8.63±5.88	8.75±5.69		9.08±5.69	8.32±5.46	8.35±5.43	8.71±5.39
Medical cure		11.24±3.03	10.02±3.49	9.93±3.25	9.95±3.28		11.27±3.46	11.29±3.33	10.72±3.91	10.89±3.71		12.25±2.80	11.21±3.38	10.92±3.14	11.07±3.12		11.57±3.29	10.55±3.57	10.69±3.56	10.88±3.42
Medication		13.44±3.79	13.06±4.04	13.15±4.03	12.73±4.34		13.38±3.32	13.44±3.41	13.21±3.36	13.08±3.43		13.96±3.73	13.75±4.11	13.40±4.25	13.21±4.30		13.05±4.53	12.49±4.96	12.37±5.01	12.32±5.12
**Patient Global Impression of Change**	15	NA	3.06±0.70	3.25±1.06	3.43±1.36	13	NA	3.15±1.21	3.06±1.48	3±0.65	24	NA	2.95±0.99	2.88±0.81	2.88±0.97	20	NA	2.80±0.83	3.14±1.31	3.47±1.16

NRS: Numerical Rating Scale; CHLC: Chance Health Locus of Control; IHLC: Internal Health Locus of Control; PHLC: Powerful Other Health Locus of Control; MCS: Mental Component Score; PCS: Physical Component Score; NA: Not Applicable.

### Effects of treatment groups over time

As depicted in Table 3, a significant time effect was found for 10 out of 19 variables. Pain intensity (p = 0.03, 95% IC [-0.179- -0.009]), insomnia severity (ISI) (p < .001, 95% IC [-0.923–-0.302]), the belief that one is disabled by pain (disability SOPA-35) (p < .001, 95% IC [-0.527–-0.169]), the belief that hurt signifies physical injury (harm SOPA-35) (p < .001, 95% IC [-0.643–-0.223]), the belief that it is the doctor’s duty to relief one’s pain (medical cure SOPA-35) (p < .001, 95% IC [-0.546–-0.197]), and the belief that medication is the best treatment for pain (medication SOPA-35) (p = 0.01, 95% IC [-0.466–-0.053) decreased significantly over time. Conversely, internal locus of health control (IHLC) (p < .01, 95% IC [0.054–0.334]), physical quality of life (PCS) (p < .001, 95% IC [0.495–1.234]), perceived control (control SOPA-35) (p < .001, 95% IC [0.342–0.742]) and the belief that emotion influences one’s pain experience (emotion SOPA-35) (p < .01, 95% IC [0.166–0.634]) significantly increased over time. No significant time effect was observed for fatigue intensity, anxiety (HADS), depression (HADS), pain disability index (PDI), chance health locus of control (CHLC), powerful other health locus of control (PHLC), mental quality of life (MCS), the belief that it is the significant other’s responsibility to assist one’s pain (solicitude SOPA-35) or the PGIC score. No group effect was observed for any variable. Furthermore, significant interactions were found for physical quality of life (PCS) (p = 0.003), the solicitude subscale of the SOPA-35 (p = 0.02), and the PGIC score (p = 0.03). No significant interaction between groups and time was found for the other outcomes. Supplementary information is available in S3 File.

**Table 3:** The effect of time on dependent variables.

	Coefficient ± SE	95% CI	p-value
**Pain Intensity (NRS)**			
Time	-0.0943 ± 0.04	[-0.179–-0.009]	0.03*
**Fatigue Intensity (NRS)**			
Time	0.003 ± 0.049	[-0.093–0.003]	0.94
**Anxiety (HADS)**			
Time	-0.046 ± 0.086	[-0.216–0.124]	0.59
**Depression (HADS)**			
Time	-0.075 ± 0.099	[-0.271–0.121]	0.45
**Insomnia Severity Index**			
Time	-0.616 ± 0.159	[-0.923–-0.302]	< .001*
**Pain Disability Index**			
Time	-0.425 ± 0.304	[-1.023–0.173]	0.16
**Chance Health Locus of Control (MHLC)**			
Time	-0.098 ± 0.073	[-0.241–0.045]	0.18
**Internal Health Locus of Control (MHLC)**			
Time	0.194 ± 0.071	[0.054–0.334]	< .01*
**Powerful Others Health Locus of Control (MHLC)**			
Time	0.006 ± 0.066	[-0.123–0.135]	0.93
**Mental Composite Score (SF-36)**			
Time	0.230 ± 0.299	[-0.358–0.819]	0.44
**Physical Composite Score (SF-36)**			
Time	0.864 ± 0.188	[0.495–1.234]	< .001*
**Control (SOPA– 35)**			
Time	0.542 ± 0.102	[0.342–0.742]	< .001*
**Disability (SOPA– 35)**			
Time	-0.348 ± 0.091	[-0.527–-0.169]	< .001*
**Harm (SOPA– 35)**			
Time	-0.433 ± 0.107	[-0.643–-0.223]	< .001*
**Emotion (SOPA– 35)**			
Time	0.400 ± 0.119	[0.166–0.634]	< .01*
**Solicitude (SOPA– 35)**			
Time	0.100 ± 0.107	[-0.109–0.309]	0.35
**Medical Cure (SOPA– 35)**			
Time	-0.381 ± 0.094	[-0.546–-0.197]	< .001*
**Medication (SOPA– 35)**			
Time	-0.259 ± 0.105	[-0.466–-0.053]	0.01*
**Patient Global Impression of Change**			
Time	-0.037 ± 0.094	[-0.225–0.150]	0.69

NRS: Numerical Rating Scale; CHLC: Change Health Locus of Control; IHLC: Internal Health Locus of Control; PHLC: Powerful Other Health Locus of Control; MCS: Mental Component Score; PCS: Physical Component Score; CI 95%: 95% Confidence Interval;

*: Significant p-value (p < .05).

Finally, multiple comparisons using Bonferroni as correction (p = 0.01) method were conducted in order to assess between which points in time patients displayed an improvement. These analyses revealed that over time there was a significant decrease in pain intensity between T1 and T3 but not between T1 and T2 or T4. A significant decrease of insomnia severity (ISI) occurred between T1 and T4 but not between T1 and T2 or T3. Similar results were observed for the disability subscale of the SOPA-35. Furthermore, significant decreases between T1 and all other time points were found for 3 subscales of the SOPA-35 (harm, emotion, and medical cure) while no significant differences were observed for the medication subscale of the SOPA-35. Significant increases were observed between T1 and all other time points for the perceived control (control subscale of the SOPA-35) as well as for the PCS subscale of the SF-36. Concerning the IHLC subscale of the MHLC, only a significant increase was found between T1 and T4. Supplementary information is available in S4 File.

## Discussion

The aim of this randomized longitudinal study was to assess whether self-hypnosis/self-care was more effective than music/self-care, self-care alone and psychoeducation/CBT. We further investigated the eventual lasting treatment effect at 6- and 12-month follow-ups. Our results showed a global effect of time for pain intensity, insomnia severity, physical quality of life, internal locus of health control and 6 subscales of the SOPA-35 (i.e., control, disability, harm, emotion, medical cure, and medication), while no group effect was observed. Significant interactions group by time were found for physical quality of life (PCS), the solicitude subscale of the SOPA-35 and the patient global impression of change (PGIC), highlighting different patterns of effect of treatments across time in the four groups. Moreover, the beneficial effects retrieved directly after the end of the treatments were maintained for both 6 and 12 months, highlighting the fact that the patients seem to benefit from treatments over one year after they had ended. Nevertheless, since adherence was low, this result has to be taken cautiously.

The present findings do not allow us to confirm our first hypothesis, i.e., the advantage of self-hypnosis combined with self-care, compared to the three other approaches. Jensen et al. [43] showed that compared to a self-hypnosis training alone or cognitive restructuring (CBT) alone, a combination of both hypnosis and CBT improved daily pain intensity, worst pain intensity, pain interference, and catastrophizing in 15 patients suffering from multiple sclerosis [43]. More recently, Castel et al. [44] randomized 93 patients suffering from fibromyalgia into three groups: standard care, CBT alone and CBT/hypnosis (14 weekly-sessions of 2 hours). Assessments were made directly after the end of the treatments and at 3- and 6-month follow-ups. Results showed that compared to standard care, patients in the CBT and CBT/hypnosis groups had improved in pain intensity, catastrophizing, psychological distress and sleep quality, and that this improvement remained at 3- and 6-month follow-ups. Furthermore, the authors highlighted the fact that the CBT effects were enhanced by the addition of hypnosis [44]. Nevertheless, and in line with our results, other authors have demonstrated otherwise. In a recent randomized study (n = 173) assessing the added value of hypnosis to CBT compared to either cognitive therapy alone or pain education in chronic pain management, the authors showed no significant differences between treatment-groups [45]. Jensen et al. [42] discussed the fact that their results could possibly stem from a similar impact on various factors inherent to care settings and common to cognitive therapy and hypnosis (i.e., motivation, expectations and therapeutic alliance). Nevertheless, even though the aim of this study was similar to the one of Jensen et al. [42], our methodology and the treatments proposed to patients are different, preventing us from relying on the same argument in order to justify our results.

In our study, the absence of a group effect could be explained by the fact that all treatment groups aimed at empowering the patients. Empowerment can be viewed as a multidimensional process through which people are able to gain control over their lives by acting on difficulties they consider essential [46]. Empowerment has various dimensions, one of them being what we aspire to foster in the patients, i.e., psychological empowerment [47]. Psychological empowerment encompasses personal control and a proactive approach to life [48]. In the specific context of health, Menon [49] proposed to define psychological health empowerment as “a cognitive state characterized by perception of control regarding one’s own health and health care; perceptions of competence regarding one’s ability to maintain good health and manage interactions with the health care system; and internalization of health ideals and goals at the individual and societal level”. According to this definition, three main characteristics of patient health empowerment can be highlighted: perceived control, perceived competence, and goal internalization [49]. This is exactly what we aimed to do in the psychoeducation/CBT groups and even more in the self-care groups. Even though the present study did not assess patients’ empowerment per se, the fact that all treatment groups significantly improved their perceived control over pain and their internal locus of control might be an indication of their empowerment.

Patient health empowerment can be considered as a patient-centered approach [50]. Although different definitions of what is a patient-centered approach are available in the literature [51], several common features can be highlighted: empathy, considering the patient’s perception and preferences, focusing on well-being, shared decision-making, giving the patients enough time to express themselves, health care remaining the patient’s responsibility while they are guided by the therapist [51–54]. Basically, the key notion of the patient-centered approach is considering that the patient is a person [55]. Another important aspect of the patient-centered approach is the communication style of the therapist [55]. This is communication that allows consideration of patients’ emotions and needs, increasing motivation and decision-making as well as promoting self-management [56,57]. Our treatment sessions were designed in the form of interactive discussions, where patients could share their experiences with one another, always accompanied by the therapist’s guidance, empathy and attentional listening. Each session aimed at enhancing the patients well-being either by explaining the interactions between pain, emotions, cognition and behavior (psychoeducation/CBT), or by only focusing on emotions, cognition and behavior (self-care). In the self-care groups (i.e., self-hypnosis/self-care, music/self-care and self-care alone), patients were given specific tasks to undertake in between sessions and could openly discuss the ease or difficulties they had in applying them. In response, the therapist gave positive feedback and empathically listened to the difficulties, enabling the patient to eventually find a way to adjust. In the psychoeducation/CBT group, even though tasks were given, they were fewer and no retroactive discussions were made. Nevertheless, the design of the psychoeducation/CBT sessions also allowed sharing of experience in between patients and the therapist.

Limitations can be addressed in this study. First, treatment adherence was relatively low as only 40 (19,7%) of the patients participated until the 12-month follow-up session. Thus, applying the ITT technique rendered our analyses robust but could not reveal a group effect because too many patients maintained the same score (sometimes the T1 score) over the 4 time points. Furthermore, in another study [58] we demonstrated that the delayed onset of treatments (≥ 4 months) and randomization significantly increased the risk of never starting the treatments. These results urged us to rethink the accuracy of only relying on randomized control trials to evaluate the effectiveness of complementary approaches. It would be interesting in future studies to propose a few sessions of pain education, for example, before the treatment, as well as other methods (e.g., qualitative, mixed methods) in order to enhance treatment adherence. A second limitation is that we did not control if patients effectively listened to the hypnosis or the music CDs on a daily basis, as recommended. Thus, it does not allow us to be sure that we effectively assessed the added value of hypnosis or music. Future studies could propose a cellphone application that would remind the patients to listen to the CD, and directly invite the patients to confirm the exercise has been done. By doing so, researchers would have access to the frequency of use of the hypnosis and/or music exercise. A third limitation is that music might have had an effect on treatment outcomes. A recent meta-analysis including 14 randomized control trials (n = 1178) has shown that music is an effective treatment in reducing pain intensity, depression and anxiety for a variety of chronic pain conditions (i.e., cancer pain, fibromyalgia, osteoarthritis, multiple sclerosis, and inflammatory bowel disease) [59]. More precisely, results indicated a moderate effect of music on pain intensity and anxiety while it yielded a high effect on depression [59]. A fourth limitation is that we did not assess patients’ empowerment. Even though their perceived control and the internal health locus of control were increased after the treatment delivery, we cannot certify that they were empowered. Inviting the patient to complete empowerment scales (e.g., the Patient Empowerment Scale [60]; the Psychological Empowerment Scale [61]) in order to assess the impact of our treatments on empowerment would be insightful. One might also argue that the diagnoses were not distributed equally among groups and that this might impact the results. We agree that according to the etiology, the treatment could have different effects. Nevertheless, the fact that we carried out univariate statistics allowed us to include, in the multivariate analyses, the diagnosis as covariates. They did not have a significant influence on the results.

In conclusion, the current study showed the beneficial effect of self-hypnosis and cognitive behavioral based approaches on the quality of life of patients with chronic pain. It seems that patients continued to apply the learned strategies as improvement was observed one year after the treatments had ended. Furthermore, poor adherence at the one-year follow-up (19.7%) highlights the difficulty of carrying out these longitudinal studies. Nevertheless, this study is encouraging as it displays the beneficial effects of complementary biopsychosocial-based treatments in chronic pain management. This is of foremost importance as the aim of the multidisciplinary approach to chronic pain management is to enable the patients to be empowered and actively participate in their well-being, even after the treatment delivery. Finally, as chronic pain represents a worldwide health problem and an economic burden, it is necessary to assess the efficiency of biopsychosocial oriented treatment that represents a low cost for the patients (a maximum of 9 sessions in our study).

## Supporting information

S1 Checklist CONSORT 2010 checklist of information to include when reporting a randomised trial*. (DOC)

S1 File Full trial protocol in French. (DOC)

S2 File Full trial protocol in English. (DOCX)

S3 File Full Table 3; Table 3: The effect of group, time, and group by time on dependent variables. (DOCX)

S4 File Table 4: Multiple comparisons between T1 (baseline) and the 3 other time points (T2; T3; T4) for scores showing significant time evolution. Adjusted p-value with Bonferroni method.
(DOCX)

S5 File Data base. (XLSX)
