# Supplementary material for: Complementary treatment comparison for chronic pain management: A randomized longitudinal study
Source: PLoS One. 2021 Aug 6;16(8):e0256001. doi: 10.1371/journal.pone.0256001 (PMC8345881; doi:10.1371/journal.pone.0256001)
Supplement: S1 File — (DOC) [file pone.0256001.s002.doc]

# DEMANDE D’AVIS AU COMITÉ D’ÉTHIQUE

# HOSPITALO-FACULTAIRE UNIVERSITAIRE DE LIÈGE

# POUR UNE ÉTUDE INTITULEE :

# Comparaison de différents traitements pour les patients souffrant de douleur chronique : une étude randomisée.

# 1. PROTOCOLE

# *1.1 TITRE DE L’ÉTUDE*

Comparaison de différents traitements pour les patients souffrant de douleur chronique : une étude randomisée.

## 1.2 POSITION DU PROBLÈME

Selon l’étude « Pain in Europe » , un adulte sur quatre en Belgique serait concerné par une problématique de douleur chronique. En 2005, les pouvoirs publics belges ont reconnu, à titre expérimental, neuf services hospitaliers, dont le service d’Algologie-Soins Palliatifs du CHU de Liège, comme centres de référence multidisciplinaires de la douleur chronique. Cette initiative s’inscrit dans le mouvement des *pain clinics* né aux États-Unis qui prônent une approche biopsychosociale de la douleur chronique et une prise en charge multidisciplinaire. L’objectif principal de notre centre est d’établir un diagnostic multidisciplinaire et de proposer un objectif thérapeutique au patient, dans une perspective biopsychosociale en vue d’identifier des relations éventuelles entre différents types de douleur (avec ou sans substrats- biomédical), des états psychologiques et des contextes de vie.

Dans une première étude, nous avons décrit le profil des patients ayant une problématique de douleur chronique qui avaient fréquenté le centre entre 2005 et 2010 . Le but de cette étude était de mieux spécifier l’expérience de la douleur chronique et de mieux comprendre les répercussions individuelles des patients, afin de mieux cibler l’orientation thérapeutique et de proposer des traitements plus adaptés. Mille huit cent trente-deux patients ont été évalués par notre équipe pluridisciplinaire comprenant médecins algologues, psychologues, kinésithérapeutes, infirmiers, assistant social et pharmacien. La durée moyenne de la douleur dans la population étudiée était de 7,5 ans, tandis que l’âge moyen était de 46,5 ans. Nous avons également observé que les femmes consultaient davantage que les hommes (81% de femmes). L’intensité moyenne de la douleur rapportée par l’ensemble des patients était de 6,1. Par ailleurs, cette étude met en évidence l’existence de deux formes d’expériences de la douleur avec des configurations biopsychosociales différentes : la première est associée aux douleurs où un substrat biomédical est perçu, est moins anxiogène et mieux vécue par le patient à la fois physiquement et mentalement ; la seconde configuration est associée à des douleurs diffuses, polymorphes sans substrat biomédical objectivable. Celle-ci est plus anxiogène pour le patient et a davantage de répercussions sur la qualité de vie. Ces résultats soulignent la nécessité d’une approche plus globale que l’approche biomédicale simple trop souvent proposée dans la prise en charge des douleurs chroniques, ainsi que la nécessité d’un travail pluridisciplinaire afin de faciliter le maintien des personnes dans des activités professionnelles, physiques et sociales qui favorisent la gestion de la douleur et contribuent positivement à leur qualité de vie.

Dans ce cadre, lors d’une seconde étude, nous avons comparé l’impact des différents traitements classiquement proposés aux patients lors de leur prise en charge dans notre centre : kinésithérapie, psycho-éducation, psycho-éducation combinée à la kinésithérapie, auto-hypnose/apprentissage de l’auto-bienveillance . En effet, actuellement peu d’études se sont intéressées à la comparaison directe de différents traitements de la douleur chronique . Dans cette étude, réalisée sur 528 patients souffrant de douleurs chroniques, non-randomisée, nous avons comparé l’effet de ces 4 types de traitement sur différentes variables telles que l’anxiété, la dépression, la qualité de vie, l’interférence de la douleur et l’intensité de la douleur. Un groupe contrôle, n’ayant reçu aucun traitement était également inclus. Les résultats démontrent un effet positif significatif de la psycho-éducation sur la composante mentale de la qualité de vie, ainsi qu’un effet positif de la psycho-éducation combinée à la kinésithérapie sur l’anxiété et l’interférence de la douleur dans le quotidien des patients. De plus, l’impact le plus important était observé chez les patients ayant été pris en charge par auto-hypnose/apprentissage de l’auto-bienveillance. Ce groupe de patients démontrent une amélioration tant au niveau de l’intensité perçue de la douleur, que de l’interférence de la douleur, l’anxiété, la dépression et la composante mentale de la qualité de vie. Le handicap perçu, le contrôle, les attentes de guérison ainsi que l’utilisation de médicaments sont significativement modifiés chez les patients impliqués dans l’apprentissage de l’auto-hypnose/auto-bienveillance .

Dans ce nouveau projet, nous aimerions pouvoir différencier l’effet de l’apprentissage de l’auto-hypnose, de l’effet de l’apprentissage des tâches d’auto-bienveillance, afin de pouvoir mettre en place dans un futur proche, une prise en charge la plus pertinente et performante possible. Pour ce faire, nous proposons à nouveau d’évaluer notre travail clinique quotidien, en ajoutant une randomisation des traitements. Les patients seraient inclus dans un des 4 groupes de traitement suivants : psychoéducation, apprentissage de l’auto-bienveillance, apprentissage de l’auto-bienveillance combiné à l’auto-hypnose, apprentissage de l’auto-bienveillance combiné à l’écoute d’une musique relaxante. La prise en charge globale du patient resterait identique à la prise en charge clinique que nous proposons actuellement. Le patient serait, dans un premier temps, vu par l’ensemble de l’équipe multidisciplinaire pour un bilan complet. Son dossier serait ensuite discuté en réunion multidisciplinaire afin de déterminer si une prise en charge de type psychosociale lui serait bénéfique. L’attribution du groupe de traitement serait faite lors de cette réunion. Après la prise en charge en groupe, le patient serait revu 6 mois et 1 an après la fin de la prise en charge, afin d’évaluer les effets retirés à court, moyen et long terme.

Certains patients viennent consulter dans notre centre avec la demande spécifique d’un apprentissage de l’auto-hypnose. Ces patients font donc preuve d’une motivation toute particulière. Nous considérons que nous ne pouvons pas les inclure dans la randomisation. Dès lors, ces patients feront partie d’un groupe auto-hypnose/auto-bienveillance, hors randomisation. Dans la seconde partie de cette étude, nous comparerons les réponses aux différents questionnaires des patients dits « motivés », aux réponses des patients ayant été inclus dans le groupe auto-hypnose/auto-bienveillance par randomisation. De la sorte, nous pourrons évaluer l’effet de la motivation sur les bénéfices retirés de ce type de prise en charge.

## 1.3 METHODE

***1.3.1. Description des différents groupes***

- **Psychoéducation** : La psychoéducation a pour objectif de responsabiliser et de pousser le patient à devenir un acteur dans sa prise en charge thérapeutique, tout en lui offrant un modèle compréhensif des mécanismes de la douleur, des bénéfices des traitements pharmacologiques, physiques et psychologiques, ainsi que des pistes pour changer sa manière de vivre au quotidien.

- **Auto-bienveillance** : Le principe de ce groupe est d’apprendre au patient à reprendre soin de lui-même au quotidien par le biais de tâches concrètes. Les objectifs sont de placer le patient dans un rôle d’acteur dans sa prise en charge, ainsi que de réactiver et d’amplifier la conscience du patient par rapport aux expériences positives rencontrées chaque jour. L’ensemble des tâches proposées sont centrées sur le bien-être général du patient plutôt que sur la problématique de la douleur. Les exercices suivants seront proposés: ajustement des attentes par rapport à soi, changer le discours que le patient à vis-à-vis de lui-même, renforcer l’estime de soi, observer et réajuster les rôles sociaux dans lesquels le patient est, identifier ses limites et ses besoins, identifier les situations pour lesquelles le patient n’a aucun pouvoir de changement, accepter l’impossibilité de tout contrôler, différencier le patient de sa douleur. Ces exercices sont expliqués et discutés en groupe pour être appliquées au quotidien. Nous demanderons au patient de tenir un journal quotidien dans lequel il écrira les tâches réalisées et les observations qu’il en retire. Nous commencerons chaque nouvelle séance par une discussion des tâches prescrites et réalisées.

- **Auto-bienveillance & auto-hypnose :** En plus des exercices décrits ci-dessus, le patient se verra proposer un exercice d’auto-hypnose de 20 minutes en fin de chaque séance. Un enregistrement de cet exercice sur CD sera donné au patient et il lui sera demandé de le pratiquer chaque jour jusqu’à la séance suivante.

**- Auto-bienveillance & musique :** En plus des exercices décrits ci-dessus, le patient se verra proposer l’écoute d’une mélodie relaxante de 20 minutes en fin de chaque séance. Cette mélodie a été composée par un musicothérapeute professionnel. Un enregistrement de cette mélodie sur CD sera donné au patient et il lui sera demandé de l’écouter chaque jour jusqu’à la séance suivante.

Chaque groupe sera composé de 8 patients. Les séances dureront 2 heures, se donneront à un rythme d’une séance par mois. Les patients suivront 9 séances au total. Les deux dernières séances auront lieu 6 mois et 1 an après le début de la prise en charge et auront pour but d’évaluer les effets retirés à plus long terme.

***1.3.2. Description des différentes étapes de l’étude***

Chaque nouveau patient rencontre, tout d’abord, un médecin algologue. Selon les répercussions du problème-douleur, l’algologue propose un bilan multidisciplinaire. Le patient rencontre ensuite une psychologue, l’équipe infirmière, l’équipe de kinésithérapeutes ou tout autre spécialiste selon la nécessité requise par le problème-douleur. Lorsque le patient a rencontré l’ensemble des intervenants, l’équipe se réunit pour discuter des observations, établir un diagnostic et proposer une approche psychosociale. La suite de la prise en charge se fait en plusieurs temps :

**- Evaluation pré-traitement (T1) :** Lors de la visite chez les infirmières, il sera demandé au patient de compléter les questionnaires suivants : Numerical Rating Scale (NRS) ; Pain Disability Index  ; Hospital Anxiety and Depression Scale  ; Short Form Health Survey questionnaire  ; Survey of Pain Attitudes – 35 ; Insomnia Severity Index [ISI –13]; Multidimensional Health Locus of Control [MHLC – 14]. Ces questionnaires font actuellement partie de la prise en charge routinière des patients venant consulter dans notre service, et pour lesquels une approche globale du problème de la douleur est préconisée lors du bilan multidisciplinaire

**- Visite chez le médecin-algologue:** Le patient est revu en consultation afin de lui expliquer les résultats de son bilan multidisciplinaire. La participation à un groupe de traitement est proposée à ce moment-là.

**- Prise en charge thérapeutique:** Le patient est intégré à un des groupes de prise en charge. L’attribution de groupe se fera de manière randomisée et aura été décidée au préalable lors de la réunion multidisciplinaire.

**- Evaluation post-traitement (T2) :** directement après la fin de la prise en charge de groupe, le patient sera recontacté afin de prendre rendez-vous pour compléter les mêmes questionnaires qu’en T0. Un questionnaire d’évaluation du changement (Patients' Global Impression of Change sera également proposé.

**- Evaluation post-traitement (T3) :** 6 mois après la fin de la prise en charge de groupe, le patient sera recontacté afin de prendre rendez-vous pour compléter les mêmes questionnaires qu’en T0. Un questionnaire d’évaluation du changement (Patients' Global Impression of Change sera également proposé.

**- Evaluation post-traitement (T4) :** 1 an après la prise en charge de groupe, le patient sera recontacté afin de prendre rendez-vous pour compléter les mêmes questionnaires qu’en T2.

## 1.4 TECHNIQUES UTILISÉES ET RISQUES POTENTIELS

## 1.4.1. Techniques utilisées

Les techniques utilisées lors de cette étude sont la psychoéducation, l’auto-hypnose et l’auto-bienveillance.

## 1.4.3 Dangers potentiels de la technique

Aucun.

## 1.5 MATÉRIEL

CDs d’auto-hypnose.

CDs – musique.

Questionnaires T1 et T2, T3, T4.

***1.6 POPULATION***

L’étude inclura 240 patients souffrant de douleur chronique et venant consulter dans notre centre d’Algologie-Soins Palliatifs (60 patients par groupe de traitement).

## 1.7 MODE DE RECRUTEMENT

La prise en charge de groupe sera proposée par l’algologue en charge du patient lors de la remise des résultats du bilan multidisciplinaire.

***1.9 CONFIDENTIALITE DES DONNEES***

Les réponses aux différents questionnaires seront encodées de manière anonyme dans une base de données accessible uniquement aux membres du personnel soignant du centre d’Algologie-Soins Palliatifs.

# Références

1. Breivik, H., et al., *Survey of chronic pain in Europe: prevalence, impact on daily life, and treatment.* Eur J Pain, 2006. **10**(4): p. 287-333.

2. Faymonville, M., et al., *The analysis of biopsychosocial characteristics of 1832 chronic pain patients consulting a tertiary pain center.* Douleur et Analgésie, 2014. **27**: p. 181-191.

3. Vanhaudenhuyse, A., et al., *Efficacy and cost-effectiveness: a study of different treatment approaches in a tertiary pain center.* European Journal of Pain, 2015. **in press**.

4. Flik, C.E., et al., *A randomised controlled trial on hypnotherapy for irritable bowel syndrome: design and methodological challenges (the IMAGINE study).* BMC Gastroenterol, 2011. **11**: p. 137.

5. Jensen, M.P., et al., *A comparison of self-hypnosis versus progressive muscle relaxation in patients with multiple sclerosis and chronic pain.* Int J Clin Exp Hypn, 2009. **57**(2): p. 198-221.

6. Miyamoto, G.C., et al., *Efficacy of the addition of modified Pilates exercises to a minimal intervention in patients with chronic low back pain: a randomized controlled trial.* Phys Ther, 2013. **93**(3): p. 310-20.

7. Toth, C., et al., *A Randomized, Single-Blind, Controlled, Parallel Assignment Study of Exercise Versus Education as Adjuvant in the Treatment of Peripheral Neuropathic Pain.* Clin J Pain, 2013.

8. Vanhaudenhuyse, A., et al., *Hypnosis and pain modulation*, in *Pain and the Conscious Brain*, L. Garcia-Larrea and P. Jackson, Editors. in press.

9. Pollard, C.A., *Preliminary validity study of the pain disability index.* Percept Mot Skills, 1984. **59**(3): p. 974.

10. Zigmond, A.S. and R.P. Snaith, *The hospital anxiety and depression scale.* Acta Psychiatr Scand, 1983. **67**(6): p. 361-70.

11. Ware, J.E., Jr. and C.D. Sherbourne, *The MOS 36-item short-form health survey (SF-36). I. Conceptual framework and item selection.* Med Care, 1992. **30**(6): p. 473-83.

12. Jensen, M., J. Turner, and J. Romano, *Pain belief assessment: A comparison of the short and long versions of the surgery of pain attitudes.* THe Journal of Pain, 2000. **1**(2): p. 138-150.

13. Bastien CH, Vallières A, Morin CM. Validation of the Insomnia Severity Index as an outcome measure for insomnia research. Sleep Med. 2001; 2:297‑307.

14. Wallston K. Multidimensional Health Locus of Control Scales. In: Gellman MD, Turner JR, éditeurs. Encyclopedia of Behavioral Medicine [Internet]. New York, NY: Springer; 2013 [cited jul 2020]. p. 1266‑1269. Available from: https://doi.org/10.1007/978-1-4419-1005-9_605

15. Hurst, H. and J. Bolton, *Assessing the clinical significance of change scores recorded on subjective outcome measures.* J Manipulative Physiol Ther, 2004. **27**(1): p. 26-35.
