# Supplementary material for: Complementary treatment comparison for chronic pain management: A randomized longitudinal study
Source: PLoS One. 2021 Aug 6;16(8):e0256001. doi: 10.1371/journal.pone.0256001 (PMC8345881; doi:10.1371/journal.pone.0256001)
Supplement: S2 File — (DOCX) [file pone.0256001.s003.docx]

**REQUEST FOR AN OPINION FROM THE ETHICS COMMITTEE**

**UNIVERSITY HOSPITAL OF LIÈGE**

**FOR A STUDY ENTITLED:**

**Comparison of different treatments for patients with chronic pain: a randomized trial**

**1. PROTOCOL**

**1.1 TITLE OF THE STUDY**

Comparison of different treatments for patients with chronic pain: a randomized trial.

**1.2 POSITION OF THE PROBLEM**

According to the "Pain in Europe" study [1], one adult in four in Belgium is affected by a chronic pain problem. In 2005, the Belgian authorities recognized, on an experimental basis, nine hospital services, including the Interdisciplinary Algology Departement of the University Hospital of Liege, as a multidisciplinary reference center for chronic pain. This initiative is in line with the movement of pain clinics born in the United States, which advocate a biopsychosocial approach to chronic pain and multidisciplinary management. The main objective of our center is to establish a multidisciplinary diagnosis and to propose a therapeutic objective to the patient, from a biopsychosocial perspective, in order to identify possible relationships between different types of pain (with or without biomedical substrates), psychological states and life contexts.

In a first study, we described the profile of patients with chronic pain problems who had attended the center between 2005 and 2010 [2]. The aim of this study was to better specify the experience of chronic pain and to better understand the individual impact of the patients, in order to better target the therapeutic orientation and to propose more adapted treatments. One thousand eight hundred and thirty-two patients were evaluated by our multidisciplinary team including algologists, psychologists, physiotherapists, nurses, social worker and pharmacist. The average duration of pain in the study population was 7.5 years, while the average age was 46.5 years. We also observed that women consulted more than men (81% women). The average pain intensity reported by all patients was 6.1. Furthermore, this study highlighted the existence of two forms of pain experience with different biopsychosocial configurations: the first is associated with pain where a biomedical substrate is perceived, is less anxiety-provoking and is better experienced by the patient both physically and mentally; the second configuration is associated with diffuse, polymorphic pain without an objectifiable biomedical substrate. The latter is more anxiety-provoking for the patient and has more repercussions on quality of life. These results underline the need for a more global approach than the simple biomedical approach too often proposed in the management of chronic pain, as well as the need for multidisciplinary work in order to facilitate the maintenance of people in professional, physical and social activities that promote pain management and contribute positively to their quality of life.

In this context, in a second study, we compared the impact of the different treatments classically offered to patients during their care in our center: physical therapy, psycho-education, psycho-education combined with physical therapy, and self-hypnosis/self-care training [3]. In fact, few studies have been conducted to directly compare different treatments for chronic pain [4-7]. In this study, carried out on 528 non-randomized chronic pain patients, we compared the effect of these 4 types of treatment on different variables such as anxiety, depression, quality of life, pain interference and pain intensity. A control group, which did not receive any treatment, was also included. The results show a significant positive effect of psycho-education on the mental component of quality of life, as well as a positive effect of psycho-education combined with physical therapy on anxiety and pain interference in patients' daily lives. Furthermore, the greatest impact was observed in patients who were managed by self-hypnosis/self-care training. This group of patients showed an improvement in both perceived pain intensity, pain interference, anxiety, depression and the mental component of quality of life. Perceived disability, control, expectations of recovery and medication use were significantly modified in patients involved in self-hypnosis/self-monitoring training [8].

In this new project, we would like to be able to differentiate the effect of learning self-hypnosis from the effect of learning self-care tasks, in order to be able to put in place in the near future the most relevant and efficient management possible. To do this, we propose to evaluate our daily clinical work again, adding a randomization of treatments. Patients would be included in one of the following 4 treatment groups: psychoeducation, self-care training, self-care training combined with self-hypnosis, self-care training combined with listening to specially composed music. The overall management of the patient would remain identical to the clinical management that we currently offer. The patient would first be seen by the whole multidisciplinary team for a complete assessment. The patient's case would then be discussed at a multidisciplinary meeting to determine whether psychosocial treatment would be beneficial. The treatment group would be assigned at this meeting. After the group treatment, the patient would be reviewed 6 months and 1 year after the end of the treatment, in order to evaluate the short, medium and long term effects.

Some patients come to our center with the specific request to learn self-hypnosis. These patients are therefore particularly motivated. We consider that we cannot include them in the randomization. Therefore, these patients will be part of a self-hypnosis/self-care group, outside the randomization. In the second part of this study, we will compare the responses to the different questionnaires of the so-called "motivated" patients with the responses of the patients who were included in the self-hypnosis/self-monitoring group by randomization. In this way, we will be able to evaluate the effect of motivation on the benefits obtained from this type of management.

**1.3 METHODS**

**1.3.1. Description of the different groups**

**- Psychoeducation**: Psychoeducation aims to empower and encourage the patient to become an actor in his therapeutic management, while offering him a comprehensive model of the mechanisms of pain, the benefits of pharmacological, physical and psychological treatments, as well as ways to change his way of living on a daily basis.

**- Self-care:** The principle of this group is to teach the patient to take care of himself on a daily basis through concrete tasks. The objectives are to place the patient in a role of actor in his care, as well as to reactivate and amplify the patient's awareness of the positive experiences encountered each day. All of the proposed tasks are focused on the patient's general well-being rather than on the problem of pain. The following exercises will be proposed: adjusting self expectations, changing the patient's self-talk, reinforcing self-esteem, observing and readjusting the social roles in which the patient is, identifying the patient's limits and needs, identifying situations in which the patient has no power to change, accepting the impossibility of controlling everything, and differentiating the patient from his pain. These exercises are explained and discussed in group to be applied in daily life. We will ask the patient to keep a daily diary in which he/she will write down the tasks performed and the observations made. We will begin each new session with a discussion of the prescribed and completed tasks.

**- Self-hypnosis combined with self-care:** In addition to the exercises described above, the patient will be offered a 20-minute self-hypnosis exercise at the end of each session. A recording of this exercise on CD will be given to the patient and he will be asked to practice it every day until the next session.

**- Specially composed music combined with self-care:** In addition to the exercises described above, the patient will be offered to listen to a relaxing melody for 20 minutes at the end of each session. This melody has been composed by a professional music therapist. A recording of this melody on CD will be given to the patient and he/she will be asked to listen to it every day until the next session.

Each group will be composed of 8 patients. The sessions will last 2 hours, and will be given at a rate of one session per month. Patients will attend a total of 9 sessions. The last two sessions will take place 1 year and 2 years after the beginning of the treatment and will aim at evaluating the effects of the treatment in the longer term.

**1.3.2. Description of the different stages of the study**

Each new patient first meets with an algologist. Depending on the impact of the pain problem, the algologist proposes a multidisciplinary assessment. The patient then meets with a psychologist, the nursing team, the team of physiotherapists or any other specialist as required by the pain problem. Once the patient has met with all the parties involved, the team meets to discuss the observations, establish a diagnosis and propose a psychosocial approach. The rest of the treatment is done in several stages:

**- Pre-treatment assessment (T1):** During the visit with the nurses, the patient will be asked to complete the following questionnaires: Numerical Rating Scale (NRS); Pain Disability Index [PDI - 9]; Hospital Anxiety and Depression Scale [HADS - 10]; Short Form Health Survey questionnaire [SF36 - 11]; Survey of Pain Attitudes - 35 [SOPA-35 - 12]; Insomnia Severity Index [ISI –13]; Multidimensional Health Locus of Control [MHLC – 14]. These questionnaires are currently part of the routine management of patients coming to our department, and for whom a global approach to the problem of pain is recommended during the multidisciplinary assessment

**- Visit to the physician-algologist:** The patient is seen again in consultation in order to explain the results of the multidisciplinary assessment. Participation in a treatment group is proposed at this time.

**- Therapeutic management:** The patient is integrated into one of the treatment groups. The group assignment will be randomized and will have been decided beforehand during the multidisciplinary meeting.

**- Post-treatment evaluation (T2):** directly after the end of the group treatment, the patient will be contacted again to make an appointment to complete the same questionnaires as in T1. A Patients' Global Impression of Change (PGIC - 15) questionnaire will also be proposed.

**- Post-treatment evaluation (T3):** 6 months after the end of the group treatment, the patient will be contacted again to make an appointment to complete the same questionnaires as in T1. A Patients' Global Impression of Change (PGIC - 15) questionnaire will also be proposed.

**- Post-treatment evaluation (T4):** 1 year after the group treatment, the patient will be contacted again to make an appointment to complete the same questionnaires as in T3.

**1.4 TECHNIQUES USED AND POTENTIAL RISKS**

**1.4.1. Techniques used**

The techniques used in this study are psychoeducation, self-hypnosis and self-monitoring.

**1.4.2 Potential dangers of the technique**

None.

**1.5 MATERIALS**

Self-hypnosis CDs.

CDs - music.

Questionnaires T1 and T1, T3, T4.

**1.6 POPULATION**

The study will include 240 patients suffering from chronic pain and coming to our Algology-Palliative Care Center (60 patients per treatment group).

**1.7 MODE OF RECRUITMENT**

The group treatment will be proposed by the algologist in charge of the patient when the results of the multidisciplinary assessment are submitted.

**1.8 CONFIDENTIALITY OF DATA**

The answers to the various questionnaires will be encoded anonymously in a database accessible only to the members of the nursing staff of the Algology-Palliative Care Center.

# REFERENCES

1. Breivik, H., et al., *Survey of chronic pain in Europe: prevalence, impact on daily life, and treatment.* Eur J Pain, 2006. **10**(4): p. 287-333.

2. Faymonville, M., et al., *The analysis of biopsychosocial characteristics of 1832 chronic pain patients consulting a tertiary pain center.* Douleur et Analgésie, 2014. **27**: p. 181-191.

3. Vanhaudenhuyse, A., et al., *Efficacy and cost-effectiveness: a study of different treatment approaches in a tertiary pain center.* European Journal of Pain, 2015. **in press**.

4. Flik, C.E., et al., *A randomised controlled trial on hypnotherapy for irritable bowel syndrome: design and methodological challenges (the IMAGINE study).* BMC Gastroenterol, 2011. **11**: p. 137.

5. Jensen, M.P., et al., *A comparison of self-hypnosis versus progressive muscle relaxation in patients with multiple sclerosis and chronic pain.* Int J Clin Exp Hypn, 2009. **57**(2): p. 198-221.

6. Miyamoto, G.C., et al., *Efficacy of the addition of modified Pilates exercises to a minimal intervention in patients with chronic low back pain: a randomized controlled trial.* Phys Ther, 2013. **93**(3): p. 310-20.

7. Toth, C., et al., *A Randomized, Single-Blind, Controlled, Parallel Assignment Study of Exercise Versus Education as Adjuvant in the Treatment of Peripheral Neuropathic Pain.* Clin J Pain, 2013.

8. Vanhaudenhuyse, A., et al., *Hypnosis and pain modulation*, in *Pain and the Conscious Brain*, L. Garcia-Larrea and P. Jackson, Editors. in press.

9. Pollard, C.A., *Preliminary validity study of the pain disability index.* Percept Mot Skills, 1984. **59**(3): p. 974.

10. Zigmond, A.S. and R.P. Snaith, *The hospital anxiety and depression scale.* Acta Psychiatr Scand, 1983. **67**(6): p. 361-70.

11. Ware, J.E., Jr. and C.D. Sherbourne, *The MOS 36-item short-form health survey (SF-36). I. Conceptual framework and item selection.* Med Care, 1992. **30**(6): p. 473-83.

12. Jensen, M., J. Turner, and J. Romano, *Pain belief assessment: A comparison of the short and long versions of the surgery of pain attitudes.* THe Journal of Pain, 2000. **1**(2): p. 138-150.

13. Bastien CH, Vallières A, Morin CM. Validation of the Insomnia Severity Index as an outcome measure for insomnia research. Sleep Med. 2001; 2:297‑307.

14. Wallston K. Multidimensional Health Locus of Control Scales. In: Gellman MD, Turner JR, éditeurs. Encyclopedia of Behavioral Medicine [Internet]. New York, NY: Springer; 2013 [cited jul 2020]. p. 1266‑1269. Available from: https://doi.org/10.1007/978-1-4419-1005-9_605

15. Hurst, H. and J. Bolton, *Assessing the clinical significance of change scores recorded on subjective outcome measures.* J Manipulative Physiol Ther, 2004. **27**(1): p. 26-35.
