# Supplementary material for: Complementary treatment comparison for chronic pain management: A randomized longitudinal study
Source: PLoS One. 2021 Aug 6;16(8):e0256001. doi: 10.1371/journal.pone.0256001 (PMC8345881; doi:10.1371/journal.pone.0256001)
Supplement: S3 File — (DOCX) [file pone.0256001.s004.docx]

**Supplementary File 3.** **Table 3**: **The effect of group, time, and group by time on dependent variables.**

|  | **Coefficient ± SE** | **IC95%** | **p-value** |
| --- | --- | --- | --- |
| **Pain Intensity (NRS)** |  |  |  |
| Time | -0.0943 ± 0.04 | [-0.179 - -0.009] | 0.03* |
| Group |  |  | 0.38 |
| Self-hypnosis/self-care vs psychoeducation/CBT | 0.0004 ± 0.30 | [-0.594 – 0.595] | 0.99 |
| Self-hypnosis/self-care vs self-care | -0.427 ± 0.308 | [-1.034 – 0.178] | 0.16 |
| Self-hypnosis/self-care vs music/self-care | 0.107 ± 0.303 | [-0.491 – 0.107] | 0.72 |
| Time*Group |  |  | 0.28 |
| Time: self-hypnosis/self-care vs psychoeducation/CBT | 0.116 ± 0.064 | [-0.008 – 0.242] | 0.07 |
| Time: self-hypnosis/self-care vs self-care | 0.052 ± 0.063 | [-0.072 – 0.175] | 0.41 |
| Time: self-hypnosis vs music/self-care | 0.019 ± 0.059 | [-0.097 – 0.137] | 0.74 |
| **Fatigue Intensity (NRS)** |  |  |  |
| Time | 0.003 ± 0.049 | [-0.093 – 0.003] | 0.94 |
| Group |  |  | 0.89 |
| Self-hypnosis/self-care vs psychoeducation/CBT | -0.158 ± 0.359 | [-0.865 – 0.548] | 0.67 |
| Self-hypnosis/self-care vs self-care | -0.234 ± 0.370 | [-0.962 – 0.493] | 0.53 |
| Self-hypnosis/self-care vs music/self-care | -0.243 ± 0.366 | [-0.966 – 0.480] | 0.51 |
| Time*Group |  |  | 0.81 |
| Time: self-hypnosis/self-care vs psychoeducation/CBT | -0.046 ± 0.072 | [-0.188 – 0.096] | 0.53 |
| Time: self-hypnosis/self-care vs self-care | -0.044 ± 0.071 | [-0.185 – 0.097] | 0.54 |
| Time: self-hypnosis vs music/self-care | 0.008 ± 0.067 | [-0.125 – 0.141] | 0.91 |
| **Anxiety (HADS)** |  |  |  |
| Time | -0.046 ± 0.086 | [-0.216 – 0.124] | 0.59 |
| Group |  |  | 0.59 |
| Self-hypnosis/self-care vs psychoeducation/CBT | 0.845 ± 0.719 | [-0.569 – 2.259] | 0.24 |
| Self-hypnosis/self-care vs self-care | 0.823 ± 0.781 | [-0.709 – 2.357] | 0.29 |
| Self-hypnosis/self-care vs music/self-care | 0.562 ± 0.794 | [-1.003 – 2.128] | 0.48 |
| Time*Group |  |  | 0.11 |
| Time: self-hypnosis/self-care vs psychoeducation/CBT | -0.296 ± 0.127 | [-0.545 - -0.046] | 0.02* |
| Time: self-hypnosis/self-care vs self-care | -0.052 ± 0.125 | [-0.298 – 0.194] | 0.68 |
| Time: self-hypnosis vs music/self-care | -0.133 ± 0.119 | [-0.366 – 0.099] | 0.26 |
| **Depression (HADS)** |  |  |  |
| Time | -0.075 ± 0.099 | [-0.271 – 0.121] | 0.45 |
| Group |  |  | 0.36 |
| Self-hypnosis/self-care vs psychoeducation/CBT | 0.791 ± 0.727 | [-0.638 – 2.219] | 0.27 |
| Self-hypnosis/self-care vs self-care | 1.275 ± 0.749 | [-0.196 – 2.747] | 0.09 |
| Self-hypnosis/self-care vs music/self-care | 0.484 ± 0.742 | [-0.978 – 1.947] | 0.51 |
| Time*Group |  |  | 0.41 |
| Time: self-hypnosis/self-care vs psychoeducation/CBT | -0.187 ± 0.146 | [-0.475 – 0.100] | 0.20 |
| Time: self-hypnosis/self-care vs self-care | -0.074 ± 0.144 | [-0.356 – 0.210] | 0.61 |
| Time: self-hypnosis vs music/self-care | -0.206 ± 0.137 | [-0.475 – 0.062] | 0.13 |
| **Insomnia Severity Index** |  |  |  |
| Time | -0.616 ± 0.159 | [-0.923 - -0.302] | <.001* |
| Group |  |  | 0.54 |
| Self-hypnosis/self-care vs psychoeducation/CBT | -1.268 ± 1.371 | [-3.960 – 1.424] | 0.35 |
| Self-hypnosis/self-care vs self-care | -2.062 ± 1.671 | [-5.358 – 1.233] | 0.22 |
| Self-hypnosis/self-care vs music/self-care | -1.883 ± 1.559 | [-4.958 – 1.193] | 0.23 |
| Time*Group |  |  | 0.09 |
| Time: self-hypnosis/self-care vs psychoeducation/CBT | 0.053 ± 0.234 | [-0.405 – 0.513] | 0.81 |
| Time: self-hypnosis/self-care vs self-care | 0.247 ± 0.233 | [-0.212 – 0.705] | 0.29 |
| Time: self-hypnosis vs music/self-care | 0.502 ± 0.219 | [0.071 – 0.933] | 0.02* |
| **Pain Disability Index** |  |  |  |
| Time | -0.425 ± 0.304 | [-1.023 – 0.173] | 0.16 |
| Group |  |  | 0.45 |
| Self-hypnosis/self-care vs psychoeducation/CBT | 1.196 ± 2.344 | [-3.408 – 5.800] | 0.61 |
| Self-hypnosis/self-care vs self-care | -2.976 ± 2.457 | [-7.802 – 1.849] | 0.23 |
| Self-hypnosis/self-care vs music/self-care | 0.440 ± 2.458 | [-4.406 – 5.287] | 0.86 |
| Time*Group |  |  | 0.64 |
| Time: self-hypnosis/self-care vs psychoeducation/CBT | -0.533 ± 0.447 | [-1.410 – 0.345] | 0.23 |
| Time: self-hypnosis/self-care vs self-care | -0.234 ± 0.442 | [-1.106 – 0.629] | 0.59 |
| Time: self-hypnosis vs music/self-care | -0.072 ± 0.417 | [-0.891 – 0.748] | 0.86 |
| **Chance Health Locus of Control (MHLC)** |  |  |  |
| Time | -0.098 ± 0.073 | [-0.241 – 0.045] | 0.18 |
| Group |  |  | 0.26 |
| Self-hypnosis/self-care vs psychoeducation/CBT | 0.964 ± 0.504 | [-0.027 – 1.954] | 0.06 |
| Self-hypnosis/self-care vs self-care | 0.687 ± 0.537 | [-0.372 – 1.746] | 0.20 |
| Self-hypnosis/self-care vs music/self-care | 0.651 ± 0.502 | [-0.339 – 1.642] | 0.19 |
| Time*Group |  |  | 0.61 |
| Time: self-hypnosis/self-care vs psychoeducation/CBT | 0.064 ± 0.107 | [-0.146 – 0.274] | 0.55 |
| Time: self-hypnosis/self-care vs self-care | 0.141 ± 0.106 | [-0.066 – 0.141] | 0.18 |
| Time: self-hypnosis vs music/self-care | 0.054 ± 0.099 | [-0.141– 0.249] | 0.59 |
| **Internal Health Locus of Control (MHLC)** |  |  |  |
| Time | 0.194 ± 0.071 | [0.054 – 0.334] | <.01* |
| Group |  |  | 0.15 |
| Self-hypnosis/self-care vs psychoeducation/CBT | 1.169 ± 0.572 | [0.047 – 2.292] | 0.04 |
| Self-hypnosis/self-care vs self-care | 0.023 ± 0.657 | [-1.273 – 1.320] | 0.97 |
| Self-hypnosis/self-care vs music/self-care | 0.725 ± 0.613 | [-0.484 – 1.934] | 0.24 |
| Time*Group |  |  | 0.90 |
| Time: self-hypnosis/self-care vs psychoeducation/CBT | -0.069 ± 0.104 | [-0.274 – 0.136] | 0.51 |
| Time: self-hypnosis/self-care vs self-care | -0.048 ± 0.103 | [-0.251 – 0.154] | 0.64 |
| Time: self-hypnosis vs music/self-care | -0.013 ± 0.097 | [-0.203 – 0.178] | 0.89 |
| **Powerful Others Health Locus of Control (MHLC)** |  |  |  |
| Time | 0.006 ± 0.066 | [-0.123 – 0.135] | 0.93 |
| Group |  |  | 0.81 |
| Self-hypnosis/self-care vs psychoeducation/CBT | 0.288 ± 0.498 | [-0.689 – 1.266] | 0.56 |
| Self-hypnosis/self-care vs self-care | -0.242 ± 0.552 | [-1.331 – 0.846] | 0.66 |
| Self-hypnosis/self-care vs music/self-care | -0.104 ± 0.515 | [-1.120 – 0.912] | 0.84 |
| Time*Group |  |  | 0.05 |
| Time: self-hypnosis/self-care vs psychoeducation/CBT | -0.156 ± 0.097 | [-0.346 – 0.034] | 0.11 |
| Time: self-hypnosis/self-care vs self-care | -0.012 ± 0.095 | [-0.200 – 0.175] | 0.89 |
| Time: self-hypnosis vs music/self-care | 0.108 ± 0.089 | [-0.068 – 0.284] | 0.23 |
| **Mental Composite Score (SF-36)** |  |  |  |
| Time | 0.230 ± 0.299 | [-0.358 – 0.819] | 0.44 |
| Group |  |  | 0.95 |
| Self-hypnosis/self-care vs psychoeducation/CBT | -0.929 ± 2.247 | [-5.342 – 3.483] | 0.67 |
| Self-hypnosis/self-care vs self-care | -1.161 ± 2.334 | [-5.745 – 3.423] | 0.62 |
| Self-hypnosis/self-care vs music/self-care | -0.200 ± 2.323 | [-4.782 – 4.380] | 0.93 |
| Time*Group |  |  | 0.60 |
| Time: self-hypnosis/self-care vs psychoeducation/CBT | 0.384 ± 0.439 | [-0.479 – 1.248] | 0.38 |
| Time: self-hypnosis/self-care vs self-care | 0.367 ± 0.435 | [-0.486 – 1.221] | 0.39 |
| Time: self-hypnosis vs music/self-care | 0.547 ± 0.412 | [-0.262 – 1.356] | 0.18 |
| **Physical Composite Score (SF-36)** |  |  |  |
| Time | 0.864 ± 0.188 | [0.495 – 1.234] | <.001* |
| Group |  |  | 0.21 |
| Self-hypnosis/self-care vs psychoeducation/CBT | 1.385 ± 1.348 | [-1.262 – 4.033] | 0.30 |
| Self-hypnosis/self-care vs self-care | 2.932 ± 1.383 | [0.215 – 5.649] | 0.03* |
| Self-hypnosis/self-care vs music/self-care | 1.262 ± 1.366 | [-1.431 – 3.956] | 0.36 |
| Time*Group |  |  | 0.003* |
| Time: self-hypnosis/self-care vs psychoeducation/CBT | -0.639 ± 0.276 | [-1.181 - -0.096] | 0.02* |
| Time: self-hypnosis/self-care vs self-care | -0.681 ± 0.273 | [-1.218 - -0.146] | 0.01* |
| Time: self-hypnosis vs music/self-care | -0.943 ± 0.258 | [-1.451 - -0.435] | <.001* |
| **Control (SOPA – 35)** |  |  |  |
| Time | 0.542 ± 0.102 | [0.342 – 0.742] | <.001* |
| Group |  |  | 0.08 |
| Self-hypnosis/self-care vs psychoeducation/CBT | -0.758 ± 0.772 | [-2.274 – 0.757] | 0.33 |
| Self-hypnosis/self-care vs self-care | -1.643 ± 0.805 | [-3.224 - -0.062] | 0.04* |
| Self-hypnosis/self-care vs music/self-care | 0.337 ± 0.803 | [-1.245 – 1.920] | 0.67 |
| Time*Group |  |  | 0.06 |
| Time: self-hypnosis/self-care vs psychoeducation/CBT | -0.345 ± 0.149 | [-0.642 - -0.056] | 0.02* |
| Time: self-hypnosis/self-care vs self-care | -0.161 ± 0.148 | [-0.451 – 0.128] | 0.27 |
| Time: self-hypnosis vs music/self-care | -0.325 ± 0.139 | [-0.599 - -0.051] | 0.02* |
| **Disability (SOPA – 35)** |  |  |  |
| Time | -0.348 ± 0.091 | [-0.527 - -0.169] | <.001* |
| Group |  |  | 0.29 |
| Self-hypnosis/self-care vs psychoeducation/CBT | -1.268 ± 0.691 | [-2.625 – 0.089] | 0.07 |
| Self-hypnosis/self-care vs self-care | -0.719 ± 0.720 | [-2.134 – 0.695] | 0.32 |
| Self-hypnosis/self-care vs music/self-care | -0.897 ± 0.718 | [-2.314 – 0.520] | 0.21 |
| Time*Group |  |  | 0.52 |
| Time: self-hypnosis/self-care vs psychoeducation/CBT | 0.181 ± 0.134 | [-0.081 – 0.444] | 0.17 |
| Time: self-hypnosis/self-care vs self-care | 0.133 ± 0.132 | [-0.126 – 0.393] | 0.31 |
| Time: self-hypnosis vs music/self-care | 0.149 ± 0.125 | [-0.095 – 0.395] | 0.23 |
| **Harm (SOPA – 35)** |  |  |  |
| Time | -0.433 ± 0.107 | [-0.643 - -0.223] | <.001* |
| Group |  |  | 0.99 |
| Self-hypnosis/self-care vs psychoeducation/CBT | 0.085 ± 0.728 | [-1.345 – 1.514] | 0.91 |
| Self-hypnosis/self-care vs self-care | 0.008 ± 0.738 | [-1.442 – 1.458] | 0.99 |
| Self-hypnosis/self-care vs music/self-care | 0.165 ± 0.723 | [-1.261 – 1.590] | 0.82 |
| Time*Group |  |  | 0.34 |
| Time: self-hypnosis/self-care vs psychoeducation/CBT | 0.173 ± 0.157 | [-0.136 – 0.481] | 0.27 |
| Time: self-hypnosis/self-care vs self-care | 0.271 ± 0.155 | [-0.034 – 0.576] | 0.08 |
| Time: self-hypnosis vs music/self-care | 0.197 ± 0.147 | [-0.091 – 0.485] | 0.18 |
| **Emotion (SOPA – 35)** |  |  |  |
| Time | 0.400 ± 0.119 | [0.166 – 0.634] | <.01* |
| Group |  |  | 0.66 |
| Self-hypnosis/self-care vs psychoeducation/CBT | -0.987 ± 0.872 | [-2.699 – 0.725] | 0.26 |
| Self-hypnosis/self-care vs self-care | -0.560 ± 0.899 | [-2.326 – 1.205] | 0.53 |
| Self-hypnosis/self-care vs music/self-care | -0.096 ± 0.890 | [-1.853 – 1.659] | 0.91 |
| Time*Group |  |  | 0.74 |
| Time: self-hypnosis/self-care vs psychoeducation/CBT | -0.160 ± 0.175 | [-0.503 – 0.183] | 0.36 |
| Time: self-hypnosis/self-care vs self-care | -0.129 ± 0.173 | [-0.469 – 0.209] | 0.45 |
| Time: self-hypnosis vs music/self-care | -0.163 ± 0.163 | [-0.483 – 0.158] | 0.32 |
| **Solicitude (SOPA – 35)** |  |  |  |
| Time | 0.100 ± 0.107 | [-0.109 – 0.309] | 0.35 |
| Group |  |  | 0.28 |
| Self-hypnosis/self-care vs psychoeducation/CBT | 1.666 ± 0.862 | [-0.028 – 3.360] | 0.05 |
| Self-hypnosis/self-care vs self-care | 0.532 ± 0.921 | [-1.278 – 2.342] | 0.56 |
| Self-hypnosis/self-care vs music/self-care | 0.411 ± 0.931 | [-1.425 – 2.247] | 0.66 |
| Time*Group |  |  | 0.01 |
| Time: self-hypnosis/self-care vs psychoeducation/CBT | -0.453 ± 0.157 | [-0.761 - -0.145] | <.01* |
| Time: self-hypnosis/self-care vs self-care | -0.019 ± 0.155 | [-0.323 – 0.285] | 0.90 |
| Time: self-hypnosis vs music/self-care | -0.208 ± 0.146 | [-0.496 – 0.079] | 0.15 |
| **Medical Cure (SOPA – 35)** |  |  |  |
| Time | -0.381 ± 0.094 | [-0.546 - -0.197] | <.001* |
| Group |  |  | 0.07 |
| Self-hypnosis/self-care vs psychoeducation/CBT | -1.675 ± 0.652 | [-2.956 - -0.396] | 0.01* |
| Self-hypnosis/self-care vs self-care | -0.710 ± 0.664 | [-2.015 – 0.594] | 0.28 |
| Self-hypnosis/self-care vs music/self-care | -1.139 ± 0.652 | [-2.456 – 0.148] | 0.08 |
| Time*Group |  |  | 0.20 |
| Time: self-hypnosis/self-care vs psychoeducation/CBT | -0.014 ± 0.137 | [-0.284 – 0.255] | 0.91 |
| Time: self-hypnosis/self-care vs self-care | 0.208 ± 0.135 | [-0.058 – 0.475] | 0.12 |
| Time: self-hypnosis vs music/self-care | 0.185 ± 0.128 | [-0.066 – 0.438] | 0.15 |
| **Medication (SOPA – 35)** |  |  |  |
| Time | -0.259 ± 0.105 | [-0.466 - -0.053] | 0.01* |
| Group |  |  | 0.14 |
| Self-hypnosis/self-care vs psychoeducation/CBT | -1.347 ± 0.774 | [-2.868 – 0.174] | 0.08 |
| Self-hypnosis/self-care vs self-care | -1.252 ± 0.799 | [-2.824 – 0.318] | 0.12 |
| Self-hypnosis/self-care vs music/self-care | -1.637 ± 0.793 | [-3.201 - -0.072] | 0.04* |
| Time*Group |  |  | 0.79 |
| Time: self-hypnosis/self-care vs psychoeducation/CBT | 0.055 ± 0.154 | [-0.248 – 0.359] | 0.72 |
| Time: self-hypnosis/self-care vs self-care | 0.147 ± 0.152 | [-0.153 – 0.447] | 0.34 |
| Time: self-hypnosis vs music/self-care | 0.029 ± 0.144 | [-0.254 – 0.313] | 0.84 |
| **Patient Global Impression of Change** |  |  |  |
| Time | -0.037 ± 0.094 | [-0.225 – 0.150] | 0.69 |
| Group |  |  | 0.44 |
| Self-hypnosis/self-care vs psychoeducation/CBT | -0.143 ± 0.439 | [-1.019 – 0.732] | 0.74 |
| Self-hypnosis/self-care vs self-care | 0.336 ± 0.445 | [-0.552 – 1.223] | 0.45 |
| Self-hypnosis/self-care vs music/self-care | -0.413 ± 0.395 | [-1.201 – 0.375] | 0.30 |
| Time*Group |  |  | 0.03* |
| Time: self-hypnosis/self-care vs psychoeducation/CBT | 0.256 ± 0.154 | [-0.048 – 0.559] | 0.09 |
| Time: self-hypnosis/self-care vs self-care | -0.039 ± 0.159 | [-0.355 – 0.275] | 0.80 |
| Time: self-hypnosis vs music/self-care | 0.352 ± 0.141 | [0.072 – 0.632] | 0.01* |

NRS: Numerical Rating Scale; CHLC: Change Health Locus of Control; IHLC: Internal Health Locus of Control; PHLC: Powerful Other Health Locus of Control; MCS: Mental Component Score; PCS: Physical Component Score; CI 95%: 95% Confidence Interval; *: significant p-value (p<.05).
