# Supplementary material for: Complementary treatment comparison for chronic pain management: A randomized longitudinal study
Source: PLoS One. 2021 Aug 6;16(8):e0256001. doi: 10.1371/journal.pone.0256001 (PMC8345881; doi:10.1371/journal.pone.0256001)
Supplement: S4 File — Adjusted p-value with Bonferroni method. (DOCX) [file pone.0256001.s005.docx]

**Supplementary File 4. Table 4:** **Multiple comparisons between T1 (baseline) and the 3 other time points (T2; T3; T4) for scores showing significant time evolution. Adjusted p-value with Bonferroni method.**

|  | **Estimate ± SE** | **Adjusted CI** | **p-value** |
| --- | --- | --- | --- |
| **Questionnaires** |  |  |  |
| **Pain Intensity (NRS)** |  |  |  |
| T1-T2  T1-T3  T1-T4 | -0.307 ± 0.136  -0.442 ± 0.159  -0.269 ± 0.155 | [-0.637 - 0.022]  [-0.87 - -0.058]  [-0.647 – 0.104] | 0.025  0.006*  0.083 |
| **Insomnia Severity Index** |  |  |  |
| T1-T2  T1-T3  T1-T4 | -0.981± 0.552  -1.365 ± 0.631  -1.945 ± 0.639 | [-2.313 – 0.351]  [-2.888 - 0.157]  [-3.490 - -0.399] | 0.077  0.032  0.003* |
| **IHLC (MHLC)** |  |  |  |
| T1-T2  T1-T3  T1-T4 | 0.274 ± 0.227  0.392 ± 0.287  0.608 ± 0.249 | [-0.274 – 0.824]  [-0.300 – 1.085]  [0.006 – 1.209] | 0.228  0.173  0.015* |
| **PCS (SF-36)** |  |  |  |
| T1-T2  T1-T3  T1-T4 | 2.242 ± 0.635  2.523 ± 0.686  2.788 ± 0.666 | [0.710 – 3.775]  [0.867 – 4.179]  [1.180 – 4.397] | <.001*  <.001*  <.001* |
| **Control (SOPA-35)** |  |  |  |
| T1-T2  T1-T3  T1-T4 | 0.923 ± 0.366  1.327 ± 0.390  1.673 ± 0.379 | [0.398 – 1.644]  [0.385 – 2.269]  [0.758 – 2.589] | 0.012*  <.001*  <.001* |
| **Disability (SOPA-35)** |  |  |  |
| T1-T2  T1-T3  T1-T4 | -0.250 ± 0.326  -0.730 ± 0.340  -1.000 ± 0.348 | [-1.037 – 0.537]  [-1.552 – 0.091]  [-1.840 - -0.160] | 0.444  0.033  0.004* |
| **Harm (SOPA-35)** |  |  |  |
| T1-T2  T1-T3  T1-T4 | -1.038 ± 0.375  -1.269 ± 0.409  -1.365 ± 0.416 | [-1.945 - -0.132]  [-2.258 - -0.280]  [-2.371 - -0.360] | 0.006*  0.002*  0.001* |
| **Emotion (SOPA-35)** |  |  |  |
| T1-T2  T1-T3  T1-T4 | 1.115 ± 0.401  1.250 ± 0.436  1.288 ± 0.466 | [0.147 – 2.083]  [0.197 – 2.303]  [0.161 – 2.416] | 0.006*  0.005*  0.006* |
| **Medical cure (SOPA-35)** |  |  |  |
| T1-T2  T1-T3  T1-T4 | -1.038 ± 0.344  -1.326 ± 0.366  -1.173 ± 0.342 | [-1.869 - -0.208]  [-2.211 - -0.442]  [-2.000 - -0.346] | 0.003*  <.001*  <.001* |
| **Medication (SOPA-35)** |  |  |  |
| T1-T2  T1-T3  T1-T4 | -0.211 ± 0.368  -0.558 ± 0.381  -0.750 ± 0.409 | [-1.101 – 0.678]  [-1.477 – 0.362]  [-1.737 – 0.237] | 0.566  0.144  0.068 |

SE: Standard Error; NRS: Numerical Rating Scale; PCS: Physical Component Score; SF-36: Short Form-36; IHLC: Internal Health Locus of Control; MHLC: Multidimensional Health Locus of Control; SOPA-35: Survey of Pain Attitudes-35; *: significant p-value (p<.01).
